# **Food-residue-level antibiotics promote mucosal colonization of foodborne antibiotic-resistant** ***Staphylococcus aureus*** **in a simulated human gut**

**DOI:** 10.1080/19490976.2025.2599517

**Published:** 2025-12-15

**Authors:** Zehua Yan, Xiaohua Zhang, Tim Fat Shum, Jiawen Xie, Jiachi Chiou, Jun Yu, Xiangdong Li

**Affiliations:** a Department of Civil and Environmental Engineering, The Hong Kong Polytechnic Universityn Hung Hom, Kowloon, Hong Kong, China; b The Hong Kong Polytechnic University Shenzhen Research Institute, Shenzhen, China; c Shenzhen Key Lab for Food Biological Safety Control, The Hong Kong Polytechnic University Shenzhen Research Institute, Shenzhen, China; d Department of Food Science and Nutrition, The Hong Kong Polytechnic Universit, Hung Hom, Kowloon, Hong Kong, China; e Research Institute for Future Food, The Hong Kong Polytechnic University, Hung Hom, Kowloon, Hong Kong, China; f Institute of Digestive Disease and Department of Medicine and Therapeutics, State Key Laboratory on Digestive Disease, Li Ka Shing Institute of Health Science, Chinese University of Hong Kong, Hong Kong, China

**Keywords:** Gut microbiota, *Staphylococcus aureus*, antibiotic resistance, antibiotic-resistant bacteria, lumen, mucosa, food-residue-level antibiotics

## Abstract

Antibiotic-resistant bacteria (ARB) and food-residue-level antibiotics in food can disrupt gut homeostasis. However, the impact of co-exposure with food-residue-level antibiotics on compartment-specific colonization dynamics and associated risks of ARB in human gut remains unclear. Here, we isolated a ciprofloxacin (CIP)-resistant *Staphylococcus aureus* strain from edible fish parts in aquaculture environment and assessed exposure risks to luminal and mucosal microbiotas using the *in vitro* Mucosal Simulator of the Human Intestinal Microbial Ecosystem (M-SHIME; ProDigest, Belgium) under three treatments: *S. aureus* alone, food-residue-level CIP alone, and co-exposure to both. Food-residue-level CIP promoted the potential colonization of *S. aureus* and relative abundance of antibiotic resistance gene hosts in the mucosal microbiota and decreased absolute abundance of 16S rRNA genes in luminal microbiota. Accordingly, microbiota exhibited compartment-specific responses: luminal microbiota exhibited increased stress tolerance potential and a tightly connected network with fewer nodes, whereas mucosal microbiota displayed enhanced resource utilization potential and a more complex network with more nodes. To investigate the mechanisms underlying these compartment-specific responses, we analyzed the microbial interconnections and enriched functions in luminal and mucosal microbiota. Notably, mucosal microbiota showed stronger positive cohesions (i.e., abundance-weighted positive correlations) within community members and enriched functions related to biofilm formation and quorum sensing, indicative of heightened communication and potential cooperation, possibly driving these compartment-specific responses. Despite these differences, continuous mucin shedding may facilitate the translocation of resistant mucosal biofilms, contributing to colonization resistance in the lumen. Our study demonstrates that food-residue-level antibiotics could facilitate *S. aureus* colonization and pose compartment-specific risks to gut microbial communities, highlighting the crucial role of intestinal mucosa for ARB colonization in human gut.

## Introduction

The ingestion of antibiotic-resistant bacteria (ARB) and antibiotic resistance genes (ARGs) through food is a significant route of human exposure, contributing to the emergence and spread of community-acquired antibiotic resistance.^1^^,^^2^ ARB may persist transiently or colonize the human gut, where they can transfer ARGs to commensal microbes via horizontal gene transfer (HGT), potentially altering microbial ecology and increasing infection risk.^3^^,^^4^ Among food sources, seafood presents a particular concern due to the widespread use of antibiotics in aquaculture and frequent detection of ARB in farmed species.^5^^,^^6^ Multiple outbreaks of gastroenteritis have been linked to ARB-contaminated seafood globally.^7^^,^^8^

Once ingested, ARB interact with the complex gut microbiota—especially in the colon,^9^ which harbors over 10^11^ microorganisms per gram.^10^^,^^11^ Some ARB may transiently or persistently colonize the gut,^3^ or potentially disseminate to extraintestinal sites,^12^ posing systemic health risks.^3^^,^^4^ Therefore, assessing the colonization potential and health risks of seafood-associated ARB is critical to understanding their contribution to antibiotic resistance dissemination.

*Staphylococcus aureus*, particularly methicillin-resistant strains (MRSA), is a clinically important ARB frequently detected in seafood.^12-14^ It has been implicated in infections of the gastrointestinal tract, lungs, skin, bloodstream, and bones.^12^^,^^14^ The human gut is considered a potential reservoir for *S. aureus*, supporting both colonization and translocation to distal tissues.^12^ Notably, *S. aureus* is asymptomatically carried by approximately one third of the healthy population,^15^ with reported levels of about 10^6^-10^7^ CFU per 100 g of feces.^16^ Despite this, foodborne *S. aureus* remains under-investigated. Prior studies using commercial antibiotic-resistant *Escherichia coli* strains in animal and *in vitro* models have revealed the colonization potential of ARB,^9^^,^^17^ but these strains often differ markedly from wild isolates in virulence, biofilm formation, and growth behavior.^18^ As a result, risk estimates based on commercial strains may not accurately reflect the health risks posed by foodborne *S. aureus*, highlighting the need to study environmentally relevant isolates.

Food-residue-level antibiotics such as ciprofloxacin (CIP), detected in aquaculture seafood at concentrations ranging from 0.01 to 100 μg/kg, may influence colonization dynamics of foodborne ARB.^19^ For example, they can disrupt microbial homeostasis, exert selective pressure^20^ and potentially increase host susceptibility to colonization by ARB.^9^^,^^21^ While prior studies have demonstrated that high^9^ or clinically relevant^22^^,^^23^ doses of antibiotics may elevate ARB colonization risks in gut, the effects of environmentally relevant co-exposure to food-residue-level antibiotics and ARB remain unclear.

Ingested ARB could colonize the intestinal lumen and mucosa,^24^ while most existing studies focus on fecal or luminal samples,^9^^,^^17^ overlooking the intestinal mucosa—a compartment with distinct microbial composition and immune functions. These structural and functional differences between the lumen and mucosa may result in compartment-specific colonization patterns and microbial responses to ARB and antibiotics. Understanding these distinctions is essential to evaluating microbial–pathogen interactions in the gut ecosystem.

**Figure 1.**
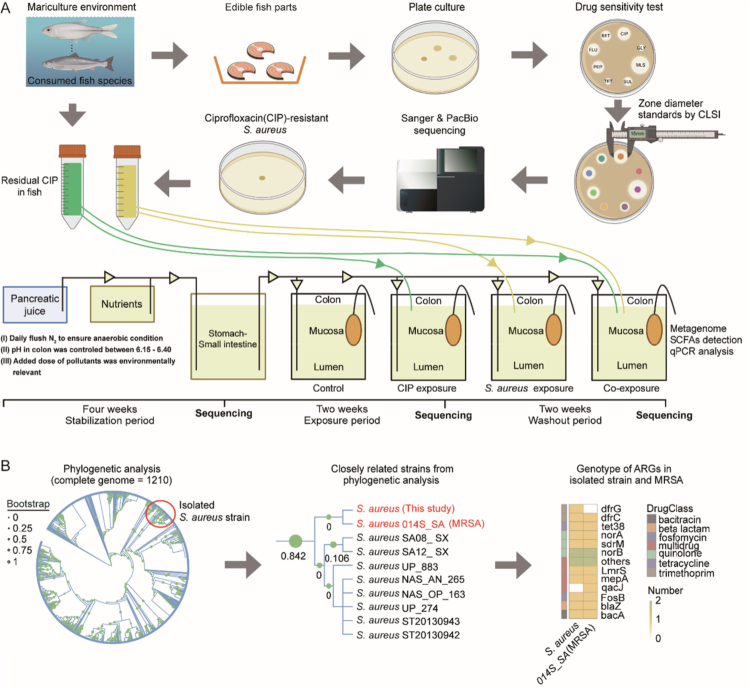
Experimental workflow of *Staphylococcus aureus* isolation and identification and mucosal simulator of the human intestinal microbial ecosystem (M-SHIME; ProDigest, Belgium) establishment. (A) Workflow for this study. First, we isolated and identified a ciprofloxacin (CIP)-resistant strain of *S. aureus* from edible fish parts in an aquaculture environment through plate culture, a drug sensitivity test, Sanger and whole genome sequencing. Second, we exposed the isolated *S. aureus* and food-residue-level CIP to the proximal colon of M-SHIME at concentrations equal to human intake from aquatic products. (B) Phylogenetic and ARG analysis of the isolated *S. aureus* strain and *S. aureus* genomes downloaded from NCBI using GTDB-Tk.

To address these knowledge gaps, we raised the following questions: (i) Does co-exposure to food-residue-level antibiotics promote colonization by antibiotic-resistant *S. aureus* in different gut compartments, and what are the associated health risks? (ii) Does exposure to food-residue-level antibiotics and antibiotic-resistant *S. aureus* alter the dynamics of antibiotic resistance carried by luminal and mucosal microbiota? and (iii) What mechanisms govern the responses of luminal and mucosal microbiota to food-residue-level antibiotics and antibiotic-resistant *S. aureus*? We hypothesized that co-exposure to food-residue-level CIP and foodborne antibiotic-resistant *S. aureus* would induce distinct colonization patterns and microbiota responses in the lumen and mucosa, driven by structural and functional differences between these compartments. To test this, we employed the Mucosal Simulator of the Human Intestinal Microbial Ecosystem (M-SHIME; ProDigest, Belgium),^25^^,^^26^ an advanced *in vitro* model that enables long-term, dynamic co-culturing of luminal and mucosal microbiota (Figure 1A). To enhance environmental relevance, we isolated a CIP-resistant *S. aureus* strain from commercial aquaculture seafood (Figure 1A).

## Materials and methods

### Fish sample collection and pathogen isolation

To investigate antibiotic-resistant pathogens in aquaculture products, four commercial fish species were collected from a mariculture farm in Hong Kong: *Siganus canaliculatus* (SC), *Trachinotus blochii* (TB), *Epinephelus coioides* (EC), and *Epinephelus fuscoguttatus × Epinephelus lanceolatus* (EFL) (Supplementary Text 1). Three adult individuals per species were sampled and stored at −20 °C within 24 h. Edible portions were plated on chromogenic agar (CHROMagar™, France) to isolate common ARB, including *Escherichia coli*, *Klebsiella*, *Staphylococcus aureus*, *Staphylococcus saprophyticus*, *Enterococcus*, *Proteus mirabilis*, and *Citrobacter*. All isolates obtained from the edible fish parts were subjected to antimicrobial susceptibility testing in accordance with the Clinical and Laboratory Standards Institute (CLSI) guidelines,^27^ using ten antibiotics spanning seven drug classes, as well as to Sanger sequencing. Based on the combined results of antimicrobial susceptibility testing and Sanger sequencing, targeted ARB were identified and subsequently selected for whole-genome sequencing using the PacBio platform. *E. coli* ATCC 25929 served as the quality control strain. Full protocols are provided in Supplementary Text 1.

### Antibiotic resistance characterization and whole genome sequencing

DNA from antibiotic-resistant isolates was extracted using the QIAamp DNA Mini Kit (Qiagen, Germany) for Sanger sequencing and whole-genome sequencing using the PacBio platform. One *S. aureus* strain resistant to 5 µg of CIP was selected, stored at −80 °C, and quantified by culturing in Luria-Bertani broth (Sigma-Aldrich, Belgium) at 37 °C followed by plate counting.^28^ Details are given in Supplementary Text 1. The genome of isolated *S. aureus* was deposited in the NCBI Sequence Read Archive (BioProject: PRJNA1347561).

### Bioreactor operation

An M-SHIME system (ProDigest, Belgium) was used to simulate the human gastrointestinal tract.^26^ The setup included five double-jacketed glass vessels: one for the stomach–small intestine and four for the proximal colon. Fecal samples were donated by a healthy volunteer without any antibiotic treatment within 6 months prior to the stool collection day.^26^^,^^29^ The collection of human fecal samples was approved by the Human Subjects Ethics Committee of The Hong Kong Polytechnic University (HSEARS20210412012). Fecal sample was homogenized in an anaerobic phosphate buffer (K₂HPO₄, KH₂PO₄, sodium thioglycolate) with sodium dithionite added prior to use. A 20% (w/w) suspension was then prepared, homogenized, and clarified by mild centrifugation to yield a reproducible fecal slurry for proximal colon vessels.

Each colon vessel was inoculated with 40 mL of fecal slurry in 500 mL of sterile nutritional medium (ProDigest, Belgium; per L: arabinogalactan 1.2 g, pectin 2 g, xylan 0.5 g, glucose 0.4 g, yeast extract 3 g, special pepton 1 g, mucin 2 g, L-cysteine 0.5 g and starch 4 g).^30^ All vessels were maintained at 37 °C under anaerobic conditions by daily nitrogen flushing and continuous stirring. Mucosal microbiotas were simulated by adding carriers (ProDigest, Belgium) coated with agar containing porcine gastric mucin (Sigma-Aldrich, Belgium).^25^ Eighty mucin-covered agar microcosms were added to mimic the mucosal environment.^25^^,^^26^ After initial static incubation, 140 mL sterile nutritional medium at pH 2 and 60 mL pancreatic juices (per L:12.5 g NaHCO_3_, 6 g dehydrated bile extract (Oxgall, Difco, USA) and 0.9 g pancreatin (Sigma-Aldrich, Belgium) per colon vessel were supplemented to the stomach-small intestine vessel: this procedure was conducted three times a day.^25^^,^^26^ Digest suspension of stomach-small intestine vessel was distributed over the different proximal colon vessels, which contained simulated colon microbiota. Colon pH was maintained at 6.15–6.40. A four-week stabilization period was conducted before treatment (Figure 1A); community stability was confirmed by short-chain fatty acid (SCFA) profiles and metagenomic sequencing. Furthermore, viable *S. aureus* was detected by plate culture after 24 hours in a sterilized digest suspension (140 mL nutritional medium at pH = 2 and 60 mL pancreatic juices), indicating that it could survive simulated gastric conditions.

After stabilization, treatments were applied for two weeks (exposure period), followed by a two-week washout period during which the CIP and *S. aureus* treatments were discontinued, while all other conditions were maintained, to assess microbiota resilience (Figure 1A). Four treatment groups were established: a control group (10 mL nutritional medium/day), an *S. aureus* group (6 × 10^5^ CFU of *S. aureus* in 10 mL nutritional medium/day), a CIP group (5 μg of CIP in 10 mL nutritional medium/day), and a co-exposure group (6 × 10⁵ CFU *S. aureus* and 5 µg CIP added separately but concurrently in 10 mL nutritional medium/day). Doses were derived from dietary intake models based on CIP and *S. aureus* concentrations in seafood,^19^^,^^31^^,^^32^ reflecting environmentally relevant human exposure levels. The calculations are provided in Supplementary Text 1. Treatments were administered directly into the lumen via sterile syringes. Luminal samples (20 mL) were collected from the suspension in the proximal colon vessel daily and stored at −20 °C; Every two days, half of the mucin-covered agar microcosms were sampled under a N_2_ flow to maintain anaerobiosis, replaced with fresh sterile units, then washed with sterile phosphate-buffered saline to remove lumen-associated bacteria, homogenized, and stored at −20 °C. Three biological replicates were used per group, with technical replicates pooled for metagenomic, quantitative polymerase chain reaction (qPCR), and SCFA analysis.

### DNA extraction and qPCR analysis

DNA was extracted from 72 luminal (1 mL) and mucosal samples (250 mg) collected during the stabilization, exposure, and washout periods using the QIAamp Fast DNA Stool Mini Kit (Qiagen, Germany). Three extraction blanks were included as quality controls. The 16S rRNA gene was quantified on a StepOnePlus Real-Time qPCR System (Applied Biosystems, USA) based on a universal primer set (Forward: TCCTACGGGAGGCAGCAGT; Reverse: GGACTACCAGGGTATCTAATCCTGTT) for the determination of bacterial load.^33^ The qPCR reaction was performed in a 20-μL system which was mixed with 10 μL Power SYBRTM Green PCR Master Mix (Thermo Fisher Scientific, USA), 0.5 μL of each primer (100 nM), 1 μL template DNA and RNase-free water to complete the final 20 µL volume.^34^ The 16S rRNA gene was amplified according to the manufacturer’s protocol of the qPCR platform: an initial step at 95 °C for 10 min for enzyme activation, then 40 cycles of 10 s at 95 °C, and 1 min at 60 °C for hybridizations and elongations, followed by a melt curve analysis in the end for specificity verification. To quantify the absolute gene copy number in the DNA samples, a seven-point standard curve (including a blank standard) in 10-fold serial dilution was run with samples for 16S rRNA gene. Triplicate samples, standards and blanks were run together in the same condition, with 90−110% amplification efficiency.

### Metagenomic sequencing and bioinformatic analysis

Sequencing was performed on an Illumina HiSeq X Ten platform, generating 2.45 Tb of high-quality reads after quality filtering. Raw reads were deposited in the NCBI Sequence Read Archive (BioProject: PRJNA1249220). Reads were used for taxonomic profiling, functional annotation, ARG identification, and the reconstruction of representative metagenome-assembled genomes (rMAGs; >90% completeness, <10% contamination). The phylogenetic relationship of the *S. aureus* isolate was conducted against NCBI RefSeq genomes (Figure 1). Bioinformatics workflows are described in Supplementary Text 2.

### Quantifying the connectivity and life-history strategy of microbial communities

To assess microbial connectivity, we calculated a cohesion index representing an abundance-weighted, zero-model corrected metric based on pairwise correlations across taxa.^35^ Higher absolute cohesion values reflect stronger correlations. Positive association indicates cooperative interactions or niche overlap, while negative association suggests competition or niche divergence. KEGG Orthology (KO) functional annotations were grouped into three life-history strategies—growth yield (Y), resource acquisition (A), and stress tolerance (S)—based on the Y-A-S framework.^36^^,^^37^ Poorly annotated functions were grouped as unclassified (U). The Y-strategy refers to the maximization microbial growth yield. The A-strategy refers to cells’ enhanced investment in gaining resources. The S-strategy refers to the ability to enhance cell tolerance under environmental stresses. Details are provided in Supplementary Text 1.

### Network construction and structure characterization

SparCC-based microbial association networks were constructed using FastSpar,^38-40^ focusing on taxa present in at least 60% of samples. Various network topological parameters, including nodes (*N*), links (L), average degree, average clustering coefficient, average path distance, centralization of degree, centralization of betweenness, density, transitivity, and modularity were calculated. The significance of empirical networks was tested against random networks. Node roles were classified based on within-module (Zi) and among-module (Pi) connectivity into module hubs, connectors, network hubs, and peripherals. We also calculated node persistence and compositional stability. Details of the network analysis are given in Supplementary Text 1.

### SCFA quantification

SCFAs (acetate, propionate, butyrate, isobutyrate, isovalerate, isocaproate) were extracted from 1 mL luminal samples using HCl and diethyl ether and detected using HPLC (Waters 515, USA) equipped with a C-18 column, UV/Visible detector (Waters 2489, USA).^41^^,^^42^

### Statistical analysis

Statistical analyses, including *α*-diversity, principal coordinates analysis (PCoA), PERMANOVA (adonis), differential abundance (edgeR), Kruskal–Wallis tests, and LEfSe, were conducted using the “vegan” (v2.6-4) and “edgeR” (v3.40) packages in R (v4.2.2). Assumptions of normality and variance homogeneity were tested prior to parametric analyzes. *P*-values were adjusted by the false discovery rate, with statistical significance defined by 95% confidence intervals (*p* < 0.05), except as indicated. Additional details are provided in Supplementary Text 3.

## Results

### Co-exposure to food-residue-level CIP enhanced *S. aureus* colonization in mucosa, inhibited growth of luminal microbiota and suppressed fermentation activity

To investigate human health risks posed by food-derived ARB, we isolated a ciprofloxacin (CIP)-resistant *Staphylococcus aureus* strain from edible fish part (Figure 1). The isolated strain was found to harbor multiple ARGs, including *mepA*, *LmrS*, and *norA*, which confer resistance to multidrug and quinolones, including CIP (Figure 1B). Phylogenetic analysis revealed a close genetic relationship (evolutionary distance = 0), indicating no sequence differences across the marker genes used by GTDB-Tk for tree analysis,^43^ as well as a similar ARG composition to the clinical methicillin-resistant strain 014S_SA (MRSA), which colonizes human skin and nasal passages^44^ (Figure 1B and Supplementary Table 1). This indicated potential health risks, so we used this strain to assess its impact on human gut microbiota.

In the M-SHIME (ProDigest, Belgium) *in vitro* gut model, four proximal colon vessels were established: control, *S. aureus* exposure only, CIP exposure only, and co-exposure (both CIP and *S. aureus*). After a stabilization period, insignificant differences were found in SCFA production (Kruskal-Wallis H-test, *p* > 0.05; Figure S1A) and microbial composition (adonis test, *p* > 0.05; Figures S1B and C). Notably, luminal and mucosal microbiota exhibited distinct compositions (adonis test, *p* < 0.05; Figure S2), dominated by Bacteroides and Firmicutes, respectively (Figure S2B) and consistent with human gut microbiota.^26^^,^^45^

**Figure 2.**
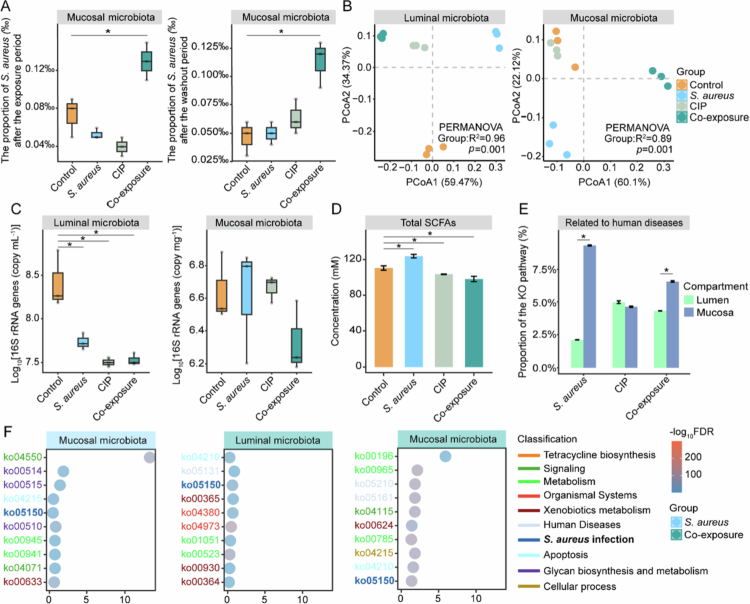
The effects of distinct treatments on the composition, putative functions, and fermentation activities of gut microbiota in the mucosal simulator of the human intestinal microbial ecosystem (M-SHIME; ProDigest, Belgium). (A) Relative abundance of *S. aureus* in mucosal microbiota at a short-read level indicated by Bracken after an exposure and a washout period in different groups, respectively. * denotes *p* < 0.05 based on the Kruskal-Wallis H-test between the corresponding treatment group and control group. (B) Principal Coordinates Analysis (PCoA) of the luminal and mucosal microbial compositions indicated by Bracken in different groups after the exposure period based on the Bray–Curtis dissimilarity. (C) Absolute abundance of 16S rRNA genes indicated by qPCR in the luminal and mucosal microbiota of different groups after the exposure period, respectively. * denotes *p* < 0.05 based on the Kruskal-Wallis H-test between the corresponding treatment group and control group. (D) Total production of short chain fatty acids (SCFAs) identified by HPLC, including isobutyrate, isovalerate, isocaproate, acetate, propionate, and butyrate in different groups after the exposure period. * denotes *p* < 0.05 based on the Kruskal-Wallis H-test between the corresponding treatment group and control group. (E) Relative abundance of KEGG Orthology (KO) pathways related to human diseases identified by eggNOG at the contig level in the luminal and mucosal microbiota of different groups after the exposure period. * denotes *p* < 0.05 based on the Kruskal-Wallis H-test between the lumen and mucosa. (F) The top 10 significantly increased KO pathways identified by eggNOG in the luminal and mucosal microbiota of the *S. aureus* and co-exposure groups based on fold change compared to the control group after the exposure period.

To evaluate *S. aureus* colonization, we analyzed its relative abundance in proximal colon vessels. Notably, in the mucosal microbiota, the relative abundance of *S. aureus* significantly increased with co-exposure to CIP, persisting even after a two-week washout period (Kruskal-Wallis H-test, *p* < 0.05; Figure 2A). However, after the exposure period, no significant change was observed in the relative abundance of *S. aureus* in luminal microbiota across all treatment groups (Figure S3). This suggests that food-residue-level CIP potentially promoted *S. aureus* colonization in mucosa.

We next evaluated how treatments influenced overall microbial composition and fermentation activities after the exposure period. Despite intrinsic differences between luminal and mucosal microbiota (adonis test, *p* < 0.05; Figure S4), treatments significantly changed the microbial community composition in both compartments (adonis test, *p* < 0.05; Figure 2B). In luminal microbiota, the absolute abundance of 16S rRNA genes significantly decreased in all treatment groups, with co-exposure exerting the strongest inhibition (Kruskal-Wallis H-test, *p* < 0.05; Figure 2C), while remaining unchanged in mucosal microbiota (Figure 2C). Similarly, in the luminal microbiota, CIP and co-exposure led to a significant decrease in the species richness (Kruskal-Wallis H-test, *p* < 0.05; Figure S5A), with co-exposure group having the lowest value; whereas, all treatments had no significant effects on it in mucosal microbiota (Kruskal-Wallis H-test, *p* > 0.05; Figure S5B). These results suggest that treatments had a stronger inhibitory effect on luminal microbiota compared to mucosal microbiota. Regarding fermentation in proximal colon vessels, co-exposure treatments significantly reduced acetic acid, propionic acid, and total SCFA production, and showed the strongest inhibitory effect on their productions; while *S. aureus* exposure significantly enhanced these productions (Kruskal-Wallis H-test, *p* < 0.05; Figure 2D and S6). These results indicate that co-exposure significantly enhanced inhibition effects on the absolute abundance of luminal microbiota and fermentation activities compared to the effects from individual *S. aureus* or CIP exposure.

### Co-exposure of food-residue-level CIP and *S. aureus* promoted putative functions related to human diseases and *S. aureus* infection in mucosa

To understand functional consequences of treatments, we identified the significantly enriched KEGG ortholog (KO) pathway in treatment groups. We first focused on enrichment of functions related to human diseases. Disease-associated functions were enriched across all treatments, with the mucosal microbiota of the *S. aureus* group exhibiting the highest proportion (9.3% of total annotated functions) (Figure 2E). In both *S. aureus* and co-exposure groups, disease-related functions were significantly higher in mucosal than luminal microbiota (Kruskal-Wallis H-test, *p* < 0.05; Figure 2E), whereas CIP exposure alone did not produce a significant compartmental difference (Kruskal-Wallis H-test, *p* > 0.05; Figure 2E). These results suggest that mucosal colonization by *S. aureus* under co-exposure conditions may increase putative functional traits linked to health risks.

To further clarify the specific enriched functions associated with health risks, the top 10 enriched pathways were identified in each treatment group relative to control group. Mucosal samples from the *S. aureus* and co-exposure groups showed notable increases in functions related to cell apoptosis and *S. aureus* infection (Figure 2F). This result further indicates *S. aureus* may colonize the intestinal mucosa.

### Co-exposure of food-residue-level CIP and *S. aureus* increased the relative abundance of ARG hosts in luminal and mucosal microbiota

**Figure 3.**
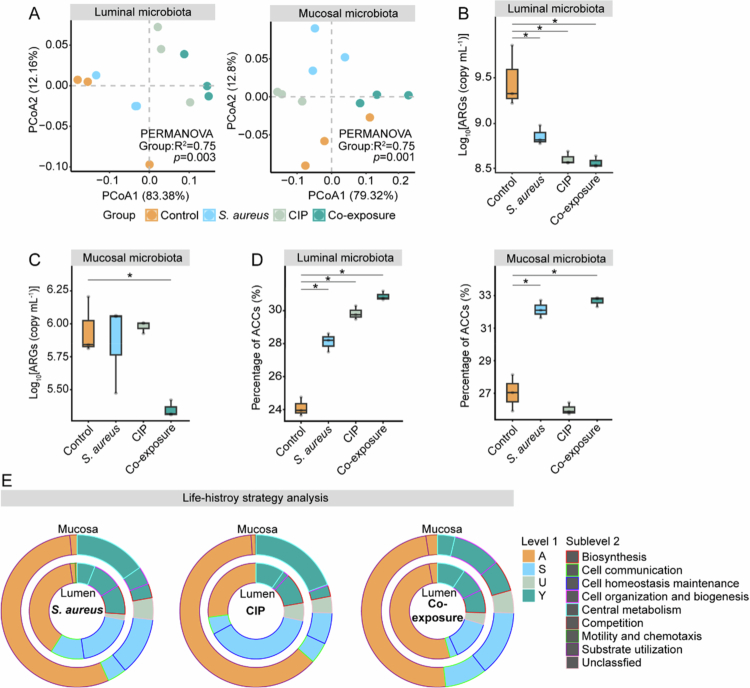
Effects of treatments on antibiotic resistance profiles and life-history strategies in the mucosal and luminal microbiota of different groups after the exposure period, respectively. (A) Principal Coordinates Analysis (PCoA) of the ARGs compositions indicated by DeepARG in luminal and mucosal microbiota of different groups after the exposure period based on the Bray–Curtis dissimilarity. (B-C) Absolute abundance of ARGs indicated by DeepARG and qPCR in the (B) luminal and (C) mucosal microbiota of different groups after the exposure period, respectively. * denotes *p* < 0.05 based on the Kruskal-Wallis H-test between the corresponding treatment group and control group. (D) Relative abundance of ARGs-carrying contigs (ACCs) indicated by MetaWrap in the luminal and mucosal microbiota of different groups after the exposure period, respectively. * denotes *p* < 0.05 based on the Kruskal-Wallis H-test between the corresponding treatment group and control group. (E) Average relative abundance of life-history strategies based on the significantly increased KO pathways indicated by eggNOG in luminal and mucosal microbiota of different treatment groups after the exposure period, respectively. The functional traits were classified into three main strategies (level 1): growth yield (Y), resource acquisition (A), and stress tolerance (S) according to the trait-based classification scheme of the Y-A-S framework and published studies.^36^^,^^37^

After the stabilization period, ARG compositions in luminal and mucosal communities were comparable across all groups (adonis test, *p* > 0.05; Figure S8). However, after a two-week exposure period, treatments significantly altered ARG compositions in luminal and mucosal microbiota (adonis test, *p* < 0.05; Figure 3A). Consistent with the 16S rRNA gene analysis, there was a significant reduction in the absolute abundance of ARGs in the luminal microbiota of all treatment groups (Kruskal-Wallis H-test, *p* < 0.05; Figure 3B). In contrast, *S. aureus* and CIP exposure did not significantly affect the abundance of ARGs in mucosal microbiota, whereas co-exposure induced a significant reduction (Kruskal-Wallis H-test, *p* < 0.05; Figure 3C).

Moreover, the relative abundance of ARG-carrying contigs (ACCs) increased significantly in both compartments under *S. aureus* and co-exposure treatments; while CIP only significantly increased it in luminal microbiota (Kruskal-Wallis H-test, *p* < 0.05; Figure 3D). In addition, the significantly increased ACC compositions were distinct across compartments and groups (Figure S9). The prevalence of ARG hosts suggests that they may be more resilient than other non-ARG-carrying contigs under *S. aureus* and co-exposure conditions.

### Divergent life-history strategies in response to treatments revealed compartment-specific adaptation

We found that the composition of putative functions (i.e., KO pathways) in luminal and mucosal microbiota varied significantly across treatment groups (adonis test, *p* < 0.05; Figure S10), suggesting distinct functional responses to each treatment. To elucidate the potential life-history strategies underlying the varied responses in different treatments and compartments, 235 enriched functions were categorized into three categories of life-history strategies: growth yield (Y)-resource acquisition (A)-stress tolerance (S) strategies (Figure 3E).

In luminal microbiota, *S. aureus* exposure enriched functions primarily associated with the A-strategy (40.93 ± 0.022%), followed by the S-strategy (31.08 ± 0.099%) (Figure 3E). In contrast, CIP exposure favored the S-strategy (44.60 ± 0.35%) over the A-strategy (27.11 ± 0.095%) (Figure 3E, Supplementary Tables 2 and 3), indicating a greater investment in countering CIP stress. In the co-exposure group, enriched traits were mainly linked to the A- (54.20 ± 0.027%) and Y- strategies (28.62 ± 0.20%) (Figure 3E).

In mucosal microbiota, the A-strategy accounted for over 50% of the enriched functions in the *S. aureus* (56.74 ± 0.0065%), CIP (63.41 ± 0.15%), and co-exposure (54.20 ± 0.027%) groups (Figure 3E). Moreover, the proportion of enriched S-strategies was higher in the luminal microbiota of the *S. aureus* (31.1 ± 0.09%) and CIP (44.6 ± 0.3%) groups compared to mucosal microbiota (16.4 ± 0.03% and 11.1 ± 0.02%; Figure 3E), indicating lower stress induction in the mucosal microbiota. Furthermore, putative biofilm formation and quorum sensing functional traits within the S-strategy increased significantly in the mucosal microbiota of the *S. aureus* and co-exposure groups (Supplementary Tables 2 and 3), indicating enhanced cell communication on the mucosa.

### Distinct ecological relationships of luminal and mucosal microbiota under treatments

**Figure 4.**
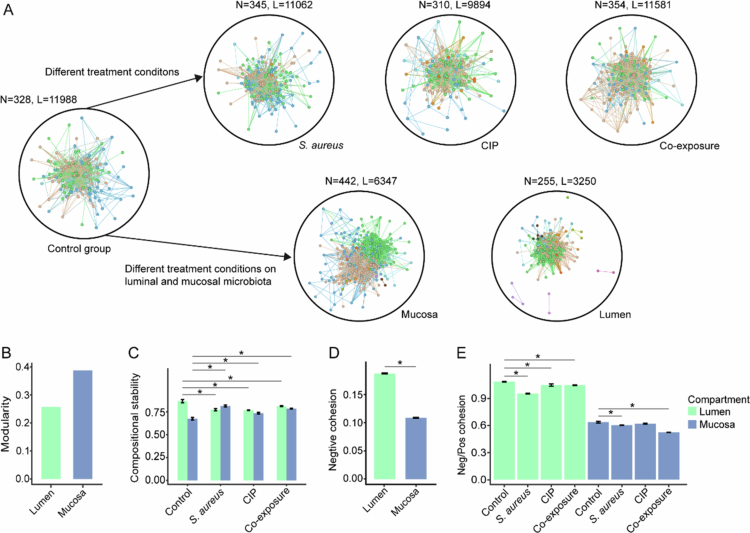
Topological parameters in the microbial networks and cohesion metric in different compartments and groups respectively. (A) SparCC networks were constructed for different compartments and treatment groups after the exposure period. N represents the nodes, and L represents the links in the networks. (B-C) (B) Modularity and (C) compositional stability in microbial networks of different compartments and groups after the exposure period. * denotes *p* < 0.05 based on the Kruskal-Wallis H-test between the corresponding treatment group and control group. (D) Absolute negative associations indicated by cohesion index reflecting negative associations in the luminal and mucosal microbiota of the control group after the exposure period. * denotes *p* < 0.05 based on the Kruskal-Wallis H-test between the lumen and mucosa. (E) The negative:positive cohesion ratio indicated by cohesion index in the gut microbiota of different compartments and treatment groups after the exposure period. * denotes *p* < 0.05 based on the Kruskal-Wallis H-test between the corresponding treatment group and control group.

Given that treatments induced strong variations in the compositions and putative functions of luminal and mucosal microbial communities, ecological relationships within these communities could also be altered. To explore these dynamics, microbial co-occurrence networks were constructed to assess ecological interactions and community stability across compartments and treatments after the exposure period (Figure 4A and Supplementary Table 4).

Network topology differed between compartments. Compared with the mucosal microbial network, which exhibited a larger size, the luminal network was smaller but more tightly connected. This was reflected in lower node numbers and shorter average path distances, but higher average degree, degree centralization, density, clustering coefficient, and betweenness centralization in luminal network (Figure 4A and S11). Topological parameters also varied among treatments. All treatment networks displayed reduced efficiency and connectivity after the exposure period, indicated by fewer links, lower clustering coefficients and transitivity, but higher average path distances (Figure 4A and S12). Furthermore, compared with the control group, networks in the *S. aureus* and co-exposure groups contained more nodes, whereas the CIP group exhibited fewer nodes (Figure 4A).

Network modularity and stability also differed between compartments. After the exposure period, modularity was lower in the luminal than in the mucosal network (Figure 4B). Moreover, relative to the control group, compositional stability of luminal microbiota declined significantly across all treatment groups (Figure 4C), while mucosal microbiota stability increased (Kruskal-Wallis H-test, *p* < 0.05; Figure 4C). In line with the 16S rRNA gene analysis, these findings indicate that the mucosal microbial community was more stable than the luminal microbiota after the exposure period. These compartment-specific responses highlight the distinct ecological interactions of luminal and mucosal microbiota under treatment conditions.

### Distinct negative/positive associations in luminal and mucosal microbiota

Given that negative and positive associations among microbial communities critically influence community stability,^46^ we then evaluated cohesion metrics after the exposure period. In the control group, the luminal microbiota exhibited a significantly higher absolute negative cohesion than the mucosal microbiota, indicating stronger negative associations in the lumen (Kruskal-Wallis H-test, *p* < 0.05; Figure 4D). After the exposure period, the ratio of negative to positive cohesion significantly decreased in both compartments of the *S. aureus* and co-exposure groups compared to the control group (Kruskal-Wallis H-test, *p* < 0.05; Figure 4E), indicating a shift toward increased positive associations. These results suggest that, compared to each other, luminal microbiota exhibited stronger negative associations, while mucosal microbiota exhibited stronger positive associations. The distinct negative and positive association in different compartments might influence their response to treatments.

### Co-increased microbial biomarkers in lumen and mucosa under *S. aureus* and co-exposure conditions

**Figure 5.**
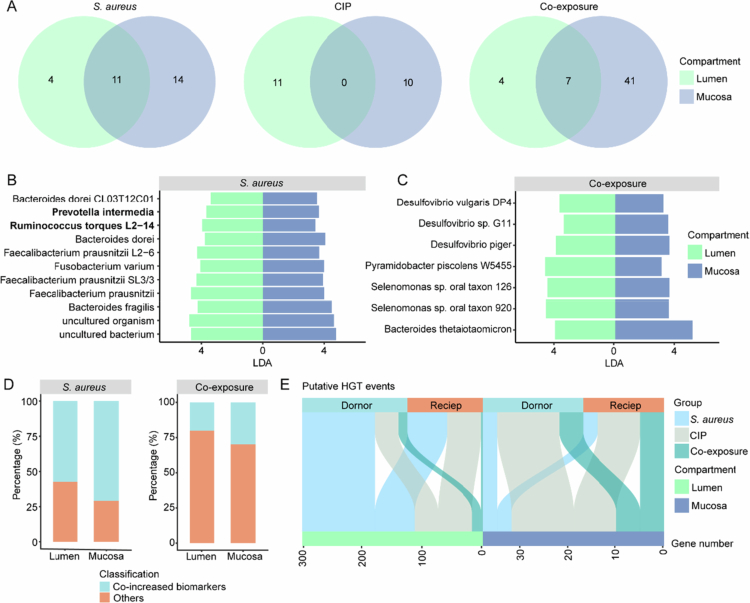
Co-increased biomarkers at the rMAG level in luminal and mucosal microbiota across treatment groups after the exposure period. (A) The number of significantly co-increased biomarkers identified by a LEfSe analysis (LDA > 3) in luminal and mucosal microbiota across treatment groups after the exposure period. (B-C) Specific co-increased biomarkers identified by a LEfSe analysis (LDA > 3) in both luminal and mucosal microbiota of (B) *S. aureus* and (C) co-exposure groups after the exposure period, respectively. Bold indicates that the species was a keystone species in a microbial network. (D) The ratio of the relative abundance of co-increased biomarkers to that of all biomarkers in the luminal and mucosal microbiota of the *S. aureus* and co-exposure groups after the exposure period. (E) The putative horizontal gene transfer (HGT) events indicated by MetaCHIP analysis of the co-increased biomarkers in luminal and mucosal microbiota across treatment groups after the exposure period.

To further investigate microbial responses, we identified significantly enriched representative metagenome-assembled genomes (rMAGs) that served as biomarkers for each treatment compared to control group in lumen and mucosa, respectively. Mucosal communities exhibited larger numbers of biomarkers in the *S. aureus* (*n* = 25) and co-exposure (*n* = 48) groups than the corresponding luminal compartment (*n* = 15 and 11, respectively; Figure 5A). This suggests that the mucosal microbiotas respond more sensitively to treatments.

Notably, 11 and 7 biomarkers co-increased in both luminal and mucosal of the *S. aureus* and co-exposure groups, accounting for 37.9% and 13.5% of all significantly increased biomarkers, respectively (Figure 5B and C). However, no co-increase was observed in luminal and mucosal microbiota of the CIP group (Figure 5A). These co-increased biomarkers included *Roseburia torques L2-14* and *Prevotella intermedia*, also acted as keystone species in the networks of the *S. aureus* group and mucosal microbiota (Figure 4A).

Despite fewer co-increased biomarkers in the co-exposure group, they accounted for a substantial proportion of the community. Specifically, co-increased biomarkers represented 57.3% and 20.1% of the total relative abundance of luminal biomarkers, and 70.8% and 29.9% of mucosal biomarkers, for *S. aureus* and co-exposure groups, respectively (Figure 5D).

To assess the broader impact of these biomarkers, horizontal gene transfer (HGT) was predicted to identify potentially transferred functional genes. Luminal biomarkers exhibited significantly more gene transfer events across all treatments (Figure 5E), particularly in the *S. aureus* group. These included genes encoding multidrug and toxin extrusion proteins in the luminal microbiota of the *S. aureus* group (Supplementary Table 5), potentially aiding detoxification. In contrast, transferred genes in the mucosal biomarkers of all treatment groups were associated with the ATP-binding proteins (e.g., K02013, K10542, K02003, K02056, K01996, K01995) (Supplementary Table 5), potentially contributing to energy acquisition by hydrolyzing ATP to power the active transport of nutrients, ions, and metabolites^47^ in mucosal environments.

### Resilience of luminal and mucosal microbiota after treatments

To assess microbial resilience after the exposure period, we analyzed the compositions and fermentation activities of gut microbiota following a two-week washout period (Figure 1). In the luminal microbiota, the absolute abundances of 16S rRNA genes remained significantly lower in the *S. aureus* and CIP groups than in the control group (Kruskal-Wallis H-test, *p* < 0.05; Figure S13A), but were comparable to those in the co-exposure group. In mucosal microbiota, the absolute abundances of 16S rRNA in all treatment groups were comparable to those in the control group after the washout period (Figure S13A). However, both the microbial and ARG compositions still differed significantly after a two-week washout period (adonis test, *p* < 0.05; Figures S13B, C and D), reflecting lingering treatment effects. Moreover, the ratio of negative to positive cohesion in both luminal and mucosal microbiota was significantly reduced in all treatment groups compared to the control group (Kruskal-Wallis H-test, *p* < 0.05; Figure S13E), indicating a shift toward increased positive associations. Notably, the luminal microbiota in the co-exposure group exhibited the lowest ratio, suggesting intensified positive associations that could facilitate recovery from greater damage. In addition, total SCFA production in all treatment groups was comparable to that in the control group (Figure S13F). These findings indicate that the mucosal microbiota was less inhibited by treatments, enabling faster recovery from inhibitory effects than the luminal microbiota.

## Discussion

This study demonstrates that food-residue-level antibiotic can promote the colonization of foodborne antibiotic-resistant *S. aureus* in the intestinal mucosa while inhibiting the growth of luminal microbiota and disrupting fermentation activity and normal functions. These findings highlight the compartment-specific vulnerability of the human gut to foodborne ARB, providing insight into how mucosal and luminal microbiotas respond differently to residual antibiotics and ARB.

### Food-residue-level CIP exposure may facilitate the colonization and infection potential of antibiotic-resistant *S. aureus* in the gut, thereby posing significant health risks

Antibiotic-resistant *S. aureus* has been frequently detected in commercial fish and seafood,^48-50^ raising concerns about foodborne infections and disruptions to gut microbial balance.^15^ Our results showed the risks and colonization of *S. aureus* may be exacerbated by food-residue-level CIP. First, food-residue-level CIP may confer a competitive advantage to CIP-resistant *S. aureus* over susceptible strains, thereby promoting its colonization.^9^^,^^19^ Additionally, CIP may destabilize gut microbial homeostasis by inducing apoptosis in taxa that otherwise inhibit *S. aureus* colonization,^51^^,^^52^ thereby weakening colonization resistance. Our results showed increase in relative abundance of *S. aureus* and putative *S. aureus*-related infection pathways within mucosal microbiota, suggesting a mucosa-specific colonization pattern of *S. aureus*, and potential host immune impairment.^53^ In contrast, all treatments significantly inhibited the growth of luminal microbiota, while mucosal microbiota remained comparatively resilient. This contrast may reflect the uniform distribution of pollutants within the lumen versus the cushioning and nutritional support provided by the mucosa.^45^^,^^54^ In addition, the reduced abundance of the 16S rRNA gene may contribute to the decline in ARG abundance within the luminal microbiota. Notably, all treatment groups exhibited a higher relative abundance of ARG hosts in the luminal compartment, highlighting the survival advantage conferred by ARGs under antibiotic pressure^55^ and heightened resistance risks. Interestingly, *S. aureus* exposure alone led to an increase in SCFA production, whereas co-exposure with CIP suppressed SCFA productions. This finding contrasts with a prior study where higher concentrations of *S. aureus* (2 × 10^10^ CFU) reduced SCFA production under short-term *in vitro* conditions.^56^ By comparison, our study applied food-residue-level doses (6 × 10^5^ CFU) over a longer duration, making direct comparisons difficult. The increase in SCFAs observed in the *S. aureus* group may be driven by enrichment of SCFA-producing taxa such as *Faecalibacterium prausnitzii*^57^. In contrast, CIP in the co-exposure group likely disrupted both community structure and fermentation activity, creating conditions that favored *S. aureus* invasion while amplifying microbiota dysregulation and therefore reducing SCFA production.

### Food-residue-level CIP exposure altered the ecological relationships of the gut microbiota

Our results demonstrated that food-residue-level CIP reduced the size, connectivity, and efficiency of microbial networks, findings consistent with observations in mouse fecal microbiota following antibiotic exposure.^58^ These alterations likely result from disruption of keystone species, which play central roles in maintaining microbial interactions^58^ or from effects on specific targeted taxa,^59^ ultimately reshaping community architecture. Beyond structural changes, CIP also reduced the stability of luminal but not mucosal networks. Previous *in vivo* study similarly reported that antibiotic exposure reduced network stability,^58^ but did not distinguish between luminal and mucosal compartments. By contrast, our *in vitro* system revealed compartment-specific responses, underscoring the importance of considering these compartments separately. In addition, CIP decreased negative associations among gut microbiota, consistent with findings from mouse studies.^58^ The decreased negative associations suggest that CIP may compromise microbial resilience^46^ and create conditions favorable for pathogen colonization.^60^ Although our study provides insights into the ecological relationships of the human gut microbiome in response to antibiotic exposure in an *in vitro* system, extrapolating these findings to the more complex *in vivo* microbial networks of the human gut remains a critical challenge for the field.

### The contrasting patterns of positive and negative associations in luminal and mucosal microbiota likely drove their compartment-specific responses to treatments

Our results indicate that luminal microbiota exhibits stronger negative associations, while mucosal microbiota exhibits stronger positive associations. As microbes compete for growth-supporting nutrients,^61^ nutrient availability likely drives these patterns. Mucosal microbes specialize in degrading mucin-derived glycoproteins,^62^ a process that often requires interspecies cooperation.^63^ The enrichment of biofilm formation and quorum-sensing pathways further suggests a greater potential for biofilm development in mucosal communities,^64^ enhancing microbial communication and cooperation.^65^ By contrast, luminal microbiota depends largely on nutrients from digested food^11^ and competes for accessible carbohydrates,^66^ fostering antagonistic (negative) interactions. This competitive environment may hinder *S. aureus* colonization in the lumen,^52^^,^^60^ where resistant commensals outcompete it for nutrients. These insights highlight the importance of studying ARB colonization specifically within the intestinal mucosa, rather than relying solely on fecal samples.

These ecological differences influence responses of luminal and mucosal microbiotas to treatments. In the mucosa, stronger positive interactions and higher modularity may promote positive feedback loops that enhance nutrient sharing but also increase vulnerability,^67^ as disruptions to one species can cascade through the community.^67^ The treatments may disrupt certain members in positive feedback loops, reducing mutual collaboration, and therefore necessitating greater A-strategy investment. In contrast, luminal communities exhibit stronger negative associations and lower modularity, favoring negative feedback loops that buffer disturbances.^68^ These dynamics reduce mutual support, requiring taxa to withstand stress independently and favoring stress-tolerant species with S-strategy traits.

### Potential translocation of biofilm-forming members from the mucosa to the lumen may further enhance microbial interactions and resistance to colonization

Notably, several co-increased microbial biomarkers were identified in both luminal and mucosal microbiota in the *S. aureus* and co-exposure groups. Many of these biomarkers are known for their ability to colonize and degrade the intestinal mucosa (e.g., *Ruminococcus torques,*^63^*Bacteroides thetaiotaomicron,*^63^*Bacteroides dorei,*^69^*Desulfovibrio vulgaris,*^70^*Desulfovibrio sp. G11* and *Desulfovibrio piger*)*.*^70^ For example, *R. torques L2−14*, a connector in the microbial network of the *S. aureus* group, degrades mucin glycoprotein and collaborates with *B. thetaiotaomicron* to access mucin-derived nutrients.^63^ The close association of co-increased biomarkers with the mucosa suggests that many luminal biomarkers may originate from ongoing mucosal shedding.^71^ Once translocated, these biofilms may promote HGT, including the dissemination of genes involved in multidrug resistance and toxin extrusion, thereby bolstering resistance to treatments. These findings alongside observed increases in positive microbial associations (indicative of cooperation), elevated A-strategy investment, and higher SCFA production, suggest that mucosal microbiota may defend against *S. aureus* colonization by fostering cooperative biofilm formation and resource sharing. Additionally, some co-increased biomarkers—such as *Prevotella intermedia*^72^ and *Faecalibacterium prausnitzii*^73^—are known to antagonize *S. aureus*. Notably, *P. intermedia* could modulate MRSA toxin expression via quorum sensing^72^ and serves as a key connector in the mucosal microbial network, underscoring its role in colonization resistance. Overall, our findings suggest that biofilms resistant to *S. aureus* likely originate in the mucosa and subsequently migrate into the lumen. These results emphasize the importance of mucosa–lumen microbial interactions in mitigating foodborne ARB colonization. Future studies should account for these cross-compartmental dynamics when assessing ARB colonization in the human gut.

### Conclusion, limitations and implications

This study demonstrates that co-exposure to food-residue-level CIP and CIP-resistant *S aureus* induces compartment-specific effects on the gut microbiota. Specifically, co-exposure promotes *S. aureus* colonization in the mucosa while inhibiting the growth of luminal communities and suppressing fermentation activity. The microbiota exhibited distinct responses across compartments, which may be induced by contrasting patterns of positive and negative ecological relationships. Despite these differences, ongoing mucin shedding may facilitate the translocation of resistant biofilm-forming microbes from the mucosa to the lumen, supporting colonization resistance and inter-compartmental connectivity. These findings highlight the critical role of the intestinal mucosa in mediating ARB colonization dynamics—an aspect often overlooked in fecal-based assessments. They also suggest that current food safety standards may underestimate the risks posed by low-level antibiotic residues in food, particularly regarding their impact on ARB colonization. While the M-SHIME (ProDigest, Belgium) model effectively simulates luminal and mucosal microbial dynamics, it does not account for host immune responses, which may influence colonization and microbial resilience. Future studies should incorporate host-microbiota interactions to more accurately assess the risks of ARB colonization under realistic exposure scenarios. In addition, although our method employed standard curves for qPCR-based gene quantification, a spike-in approach would improve the robustness of absolute abundance measurements. Future research should incorporate this method to enable more precise quantification of specific genes and a better understanding of microbial response. Furthermore, we used a widely applied method for predicting functions from metagenomic data; however, this approach may lead to over-interpretation of certain genes.^74^ Future metagenomic studies should interpret such functions with caution or validate key predicted functions through complementary approaches such as transcriptomics. Overall, this work identifies the intestinal mucosa as a key site in ARB colonization and underscores the need for compartment-specific strategies in surveillance, risk assessment, and microbiome-targeted interventions.

## Supplementary Material

Supplementary Material MSHIMESI20251029

## Data Availability

All of the raw sequencing data have been deposited in the NCBI Sequence Read Archive (BioProject accession number: PRJNA1249220). All other data supporting the findings of this study are available within the main text or supplementary materials.
