# Supplementary material for: Food-residue-level antibiotics promote mucosal colonization of foodborne antibiotic-resistant Staphylococcus aureus in a simulated human gut
Source: Gut Microbes. 2025 Dec 15;17(1):2599517. doi: 10.1080/19490976.2025.2599517 (PMC12710920; doi:10.1080/19490976.2025.2599517)
Supplement: Supplementary Material — MSHIMESI20251029 [file KGMI_A_2599517_SM2049.docx]

**Food-Residue-Level antibiotics promote mucosal colonization of foodborne antibiotic-resistant *Staphylococcus aureus* in a simulated human gut**

Zehua Yan^1,2^, Xiaohua Zhang^1,2^, Shum Tim Fat^3,4^, Jiawen Xie^1^, Jiachi Chiou^3,4,5^, Jun Yu^6^, Xiangdong Li^1,2^*

*^1^Department of Civil and Environmental Engineering, The Hong Kong Polytechnic University, Hung Hom, Kowloon, Hong Kong, China*

*^2^The Hong Kong Polytechnic University Shenzhen Research Institute, Shenzhen 518057, China*

^3^*Shenzhen Key Lab for Food Biological Safety Control, The Hong Kong Polytechnic University Shenzhen Research Institute, Shenzhen 518000, China*

*^4^Department of Food Science and Nutrition, The Hong Kong Polytechnic University, Hung Hom, Kowloon, Hong Kong, China*

*^5^Research Institute for Future Food, The Hong Kong Polytechnic University, Hung Hom, Kowloon, Hong Kong, China*

*^6^Institute of Digestive Disease, Li Ka Shing Institute of Health Science, Shenzhen Research Institute, State Key Laboratory on Digestive Disease, Chinese University of Hong Kong, Hong Kong, China*

Table of contents

**Supplementary Text**

[Supplementary Text 1. Supplementary materials and methods S4](#_Toc211963290)

[Isolation, plate culture, antimicrobial resistance assessment S4](#_Toc211963291)

[Sanger, whole-genome sequencing and quantification of antibiotic-resistant isolates S4](#_Toc211963292)

[Quantify daily exposure dose of foodborne residual ciprofloxacin and *S. aureus* S5](#_Toc211963293)

[Quantify the connectivity and life-history strategy of microbial communities S5](#_Toc211963294)

[Network construction and structure characterization S6](#_Toc211963295)

[Supplementary Text 2. Metagenomic sequencing and bioinformatic analysis S8](#_Toc211963296)

[ARGs identification and taxonomic annotation based on short reads S8](#_Toc211963297)

[Metagenomic assembly, functional and taxonomic annotation S8](#_Toc211963298)

[Identification of ARG hosts and potential HGT events S9](#_Toc211963299)

[Supplementary Text 3. Data analysis and statistics S10](#_Toc211963300)

**Supplementary Figures**

[Fig. S1 The microbial compositions and metabolic activities in different groups after the stabilization period. S11](#_Toc212405183)

[Fig. S2 The compositions of luminal and mucosal microbiota in all groups after the stabilization period. S12](#_Toc212405184)

[Fig. S3 The relative abundance of *S. aureus* in luminal microbiota at short-read level after the (A) exposure and (B) washout period, respectively. S13](#_Toc212405185)

[Fig. S4 The Principal Coordinates Analysis (PCoA) analysis of the luminal and mucosal microbial compositions in different groups after the exposure period based on the Bray–Curtis dissimilarity. S14](#_Toc212405186)

[Fig. S5 The species richness of (A) luminal and (B) mucosal microbiota in different groups after the exposure period. S15](#_Toc212405187)

[Fig. S6 Production of (A) acetate and (B) propionate in different groups after the exposure period. S16](#_Toc212405188)

[Fig. S7 The significantly increased top 10 KO pathways in (A) luminal microbiota of *S. aureus* group, and (B) luminal and (C) mucosal microbiota of CIP group based on the fold change compared to the control group after the exposure period. S17](#_Toc212405189)

[Fig. S8 The Principal Coordinates Analysis (PCoA) analysis of the ARGs composition in (A) luminal and (B) mucosal microbiota in different groups after the exposure period based on the Bray–Curtis dissimilarity. S18](#_Toc212405190)

[Fig. S9 The average relative abundance of genera in the significantly increased ARG-carrying contigs (ACCs) of (A) *S. aureus*, (B) CIP and (C) co-exposure groups after the exposure period. S19](#_Toc212405191)

[Fig. S10 The Principal Coordinates Analysis (PCoA) analysis of the composition of KEGG Orthology (KO) pathways in contigs of (A) luminal and (B) mucosal microbiota in different groups after the exposure period based on the Bray–Curtis dissimilarity. S20](#_Toc212405192)

[Fig. S11 Topological parameters in the microbial networks of luminal and mucosal microbiota S21](#_Toc212405193)

[Fig. S12 Topological parameters in the microbial networks of different groups after the exposure period, respectively. The (A) total nodes, (B) total links, (C) average clustering coefficient (avgCC), (D) average path distance (GD) and (E) transitivity (Trans) in the microbial networks of different groups after the exposure period, respectively. S22](#_Toc212405194)

[Fig. S13 The microbial compositions, antibiotic resistance profiles, and fermentation activities of gut microbiota in different groups after the washout period. S23](#_Toc212405195)

**Supplementary Tables**

[Supplementary Table 1. The information of the close-related complete genome with the isolated *S. aureus* from NCBI (https://www.ncbi.nlm.nih.gov/pathogens) S25](#_Toc212405196)

[Supplementary Table 2. The proportion of life-history strategies in each group and the proportion of level 2 classification in them S26](#_Toc212405197)

[Supplementary Table 3. Classification of life-history strategies based on KO pathways ^11, 12^ S27](#_Toc212405198)

[Supplementary Table 4. Topological properties of the networks in different compartments and groups after exposure period. S36](#_Toc212405199)

# Supplementary Text 1. Supplementary materials and methods

**Fish sample collection**

To investigate the emergence of antibiotic-resistant pathogens in cultured seafood, several commercially farmed fish species from a typical mariculture facility in Hong Kong were selected for ARB analysis. The farm is located on the eastern coast of Hong Kong near Tai Tau Chau Island (22.36865, 114.32309), far from urban areas and influenced by clean seawater from the South China Sea ^1^. Thus, pollution in this mariculture environment primarily originates from farming activities Commonly consumed fish species, including *Siganus canaliculatus* (Park, 1797; SC, herbivore), *Trachinotus blochii* (Lacepède, 1801; TB, omnivore), *Epinephelus coioides* (Hamilton, 1822; EC, carnivore), and *Epinephelus fuscoguttatus x Epinephelus lanceolatus* (EFL, carnivore), are cultured in this farm. In May 2020, three adult individuals of each species of similar size were sampled. To prevent contamination, fishing nets were rinsed with on-site seawater before use. After capture, each fish was placed in a sterilized plastic bag for immediate storage. All samples were preserved in containers filled with freezer packs at 4 °C and transported to the laboratory within 24 hours, where they were stored at -20 °C.

## Isolation, plate culture, antimicrobial resistance assessment

The edible parts (including skin and muscle) of each fish species were dissected, pooled, and homogenized in sterile blenders. A 2.5-gram portion of the homogenized sample was incubated in liquid LB broth for 24 hours at 37 °C. Subsequently, 10 µl of each sample was evenly spread onto a solid chromogenic medium plate (CHROMagar™, France) for the isolation and differentiation of pathogens, including *Escherichia coli*, *Klebsiella*, *Staphylococcus aureus*, *Staphylococcus saprophyticus*, *Enterococcus*, *Proteus mirabilis*, and *Citrobacter*. Overlapping colonies were further processed using a 10-fold serial dilution to obtain individual colonies. Each isolated colony underwent antimicrobial susceptibility testing via the disk-diffusion method according to Clinical and Laboratory Standards Institute (CLSI) guidelines ^2^. Ten antimicrobial disks from seven drug classes were selected, including beta-lactam (ceftriaxone, 30 µg; imipenem, 10 µg; cefotaxime, 30 µg; ampicillin, 10 µg), fluoroquinolone (ciprofloxacin, 5 µg), peptides (polymyxin B, 300 IU), glycopeptide (vancomycin, 30 µg), tetracycline (tetracycline, 30 ug), sulfonamide (trimethoprim-sulfamethoxazole, 1.25 - 23.75 µg), macrolide-lincosamide-streptogramin (MLS; erythromycin, 15 µg). The *E. coli* ATCC 25929 was used as a quality control strain throughout the testing. The isolates were classified as susceptible, intermediate, or resistant according to CLSI zone diameter interpretative standards. All culture experiments were performed in triplicate for each fish species.

## Sanger, whole-genome sequencing and quantification of antibiotic-resistant isolates

Total genomic DNA from each antibiotic-resistant isolate was extracted using the QIAamp DNA Mini Kit following the manufacturer’s protocol and initially sequenced by Sanger sequencing. The taxonomy of each isolate was annotated by blasting the sequence on the NCBI platform. If an isolate belonged to a target pathogen, PacBio sequencing was conducted to obtain whole genome information. Briefly, after quality control for concentration, integrity, and purity, DNA was sheared using Covaris g-TUBEs to an average size of 7–10 Kb. The DNA fragments then underwent single-strand overhang removal, damage repair, and end repair. Subsequently, the DNA repaired from each bacterial strain was ligated with SMRTbell adapters with distinct barcodes to form SMRTbell sequencing libraries. Following purification with 0.45x AMPure PB beads, the eight sequencing libraries were pooled according to PacBio’s Microbial Multiplexing Calculator. The pooled library was sequenced on the PacBio Sequel platform. Raw sequencing reads were demultiplexed and assembled using SMRT Link software (v7.0). Genome assembly was performed with the Assembly (HGAP 4) application in SMRT Link using default settings. Ultimately, a CIP-resistant strain of *S. aureus* was isolated and stored at -80 °C.

To quantify the isolated *S. aureus*, the stored strain was transferred to LB broth medium for overnight incubation at 37 °C. The optical density (OD) at 600 nm (OD600) was measured using a spectrophotometer. Colony-forming units (CFU) per ml were then determined by plating 100 μl aliquots of *S. aureus* cultures, diluted in 0.9% NaCl, onto LB agar plates following standard plate counting methods ^3^. The calculated CFU of *S. aureus* was used in subsequent exposure experiments.

## Quantify daily exposure dose of foodborne residual ciprofloxacin and *S. aureus*

The daily intake (DI) model was used to quantify daily exposure to residual ciprofloxacin (CIP) and *S. aureus* in fish from aquaculture environments ^4^. First, the average fish consumption per person per day was adopted from a previous study as 59.3 g ^5^. In addition, reported residual levels of antibiotics in aquatic products range from 0.01 to 100 μg/kg wet weight (ww), with CIP from the quinolone class frequently detected ^6^. Therefore, we used CIP as a representative antibiotic in foodborne residues and calculated the maximum daily residual antibiotic exposure to be 5.93 μg/day per person using Equation (1). Drug sensitivity testing confirmed that the isolated *S. aureus* strain was resistant to CIP at 5 μg; thus, we set the daily exposure dose of CIP to 5 μg to reflect environmental relevance and resistance of *S. aureus*. Additionally, the average concentration of *S. aureus* in fish samples can reach up to 1 × 10^4^ CFU/g ^7^. Consequently, the daily exposure dose of *S. aureus* was set as 6 × 10^5^ CFU based on Equation (2).

DI of residual CIP (μg/day) = concentration of antibiotics in fish (μg/g) × consumption quantity of fish (g/day) (1) ^4^

DI of residual *S. aureus* (CFU/day) = concentration of *S. aureus* in fish (CFU/g) × consumption quantity of fish (g/day) (2) ^4^

## Quantify the connectivity and life-history strategy of microbial communities

To assess the connectivity of gut microbiota in response to pollutant exposure, we used the cohesion index ^8^, an abundance-weighted, zero-model corrected metric based on taxon correlations across samples. Cohesion values range from −1 to 1, with negative cohesion values (−1 to 0) indicating competitive interactions and positive values (0 to 1) indicating cooperative interactions. Higher absolute cohesion values reflect stronger correlations. Positive cohesion reflects high niche overlap and/or positive interactions between taxa (potentially indicating cooperation), whereas negative cohesion indicates divergent niches and/or negative interactions (potentially indicating competition). The ratio of negative:positive associations can impact community stability.

Additionally, KEGG Orthology (KO) pathways in different groups were analyzed based on previous studies ^9, 10^ (Details in Supplementary Text 2). Subsequently, the KO pathways were first grouped into their ancestral classes, which were referred to as the functional sublevels (levels 2) according to hierarchical classifications from public databases (*e.g.*, Kyoto Encyclopedia of Genes and Genomes) or the previously published literature ^11, 12^. These functional sublevels were then classified into three main strategies (level 1): growth yield (Y), resource acquisition (A), and stress tolerance (S) based on the Y-A-S framework as previously defined ^12^. The Y-A-S framework reflects microbial life-history strategies under varying resource availability and environmental stress. The Y-strategy refers to the maximization of microbial growth yield, which includes the functional sublevels for enhancing central metabolism and biosynthesis. The A-strategy refers to cells’ enhanced investment in gaining resources, which includes the functional sublevels for improving motility, competition, substrate transportation, and biodegradation. The S-strategy refers to the capability to enhance cell tolerance under environmental stresses, which includes the functional sublevels such as sporulation, biomolecular damage repair, and osmotic protection. Functions not clearly classified were grouped under the unclassified (U) strategy. Classification results were confirmed by cross-referencing key functional traits with the microbial trait database used for Y-A-S strategy classification ^11, 12^.

## Network construction and structure characterization

To investigate the interconnections of gut microbiota in response to pollutant exposure, SparCC networks at the contig level were constructed using FastSpar ^13^. SparCC is an algorithm that accounts for sparse compositional data in network construction and is more robust than traditional correlation analyses ^14, 15^. A total of 6 networks (4 groups + 2 microbial compartments) were constructed after the exposure period, enabling comparisons of network structures across different treatments and microbial compartments. To ensure reliability, only species present in 80% of the samples were included. The 100 permutations were used to calculate the significance of correlations between species abundances and the correlation matrices with correlations > 0.6 and significance < 0.05 for subsequent network construction. Networks were visualized using the R package igraph, and topological parameters, including the number of nodes, links, average degree, clustering coefficient, path distance, degree centralization, betweenness centralization, and density, were assessed. Additionally, network modularity, measuring the extent to which a network is divided into highly connected modules with few external links, was evaluated. To test the significance of the constructed empirical networks, random networks corresponding to each empirical network were constructed by keeping the numbers of nodes and links constant and rewiring the nodes. The means and standard deviations of network topological properties from the 100 randomizations were calculated and compared with those from the corresponding empirical networks.

Each node’s topological role was classified based on within-module connectivity (Zi) and among-module connectivity (Pi) ^16^. The keystone species, that is, identified module hubs (Zi ≥ 2.5, Pi < 0.62), connectors (Zi < 2.5, Pi ≥ 0.62), and network hubs (Zi ≥ 2.5, Pi > 0.62), however all other nodes were categorized as peripherals ^15, 16^. We calculated the compositional stability for microbial communities in the networks, which indicated the change in community structure over time ^16^. Higher value of compositional stability indicates microbial community composition became more stable.

# Supplementary Text 2. Metagenomic sequencing and bioinformatic analysis

## ARGs identification and taxonomic annotation based on short reads

The analysis of ARGs was performed on metagenomes from a total of 72 samples. These comprised both luminal and mucosal samples collected at different time points from the proximal colon vessels of the Mucosal Simulator of the Human Intestinal Microbial Ecosystem (M-SHIME; ProDigest, Belgium) system. After trimming sequencing adaptors and filtering low-quality reads using fastp (v0.21.0) with default parameters ^17^, 72 samples with 935 Gb of high-quality data were obtained in total. Taxonomic classification was performed using Kraken2 (v2.1.2) with the k2_standard_20210517_38GB database ^18^and the relative abundance of species was determined using Bracken (v2.6.2) ^19^. The DeepARG (v2.0) short-read pipeline (identity = 70%, probability = 0.8, E-value = 1e−10) was used to predict the ARGs and the relative abundance of identified ARGs was normalized to the sequence number of the 16S rRNA gene ^20^.

## Metagenomic assembly, functional and taxonomic annotation

To understand the health risks of the isolated *S. aureus*, 1,210 complete genomes of *S. aureus* were downloaded from NCBI (https://www.ncbi.nlm.nih.gov/) using the NCBI Genome database ^21^. The genome set of a *S. aureus* was annotated using the *gtdbtk classify* and *gtdbtk infer* to identify the evolutionary relationships between different genomes. The evolutionary relationships of the isolated *S. aureus* were determined by comparing them with those in the evolutionarily closest genomes (<https://www.ncbi.nlm.nih.gov/pathogens>).

The filtered clean reads by fastp from all collected luminal and mucosal samples in proximal colon vessels were assembled using MetaWrap (v1.2.1) with default parameters ^22^, and the resulting individually assembled contigs (≥1000 bp) were annotated using Centrifuge (v 1.0.4) ^23^ with default parameters. To retrieve metagenome-assembled genomes (MAGs), the co-assembled contigs were clustered using three methods: MetaBAT2 (v2.12.1), Maxbin2 (v2.2.6), and Concoct (v1.0.0) ^24^. The resulting three bin sets were consolidated and filtered with the Bin_refinement module. The completeness and contamination of each bin from this bin set were evaluated using CheckM (v1.0.12) ^24, 25^. Bins with ≥90% completeness and ≤10% contamination were retained and dereplicated using dRep (v2.6.2) as representative metagenome-assembled genomes (rMAGs) ^26^. The taxonomy of rMAGs was annotated with the GTDB-Tk (v2.1.1) and their abundances were determined using coverM (v0.6.1). The genome of the isolated *S. aureus* strain, assembled contigs (≥1000 bp) and rMAGs were used to predict open reading frames (ORFs) using Prodigal (v2.6.3) ^27^. The ORF sequences were dereplicated and clustered at 90% identity using CD-HIT (v4.6) ^28^. The clustered ORF sequences were assigned putative function predictions using eggNOG-mapper ^10^.

## Identification of ARG hosts and potential HGT events

The clustered ORF sequences were searched against the ResFam database using the hmmscan function of HMMER (v3.1b2) with the –cut_ga parameter ^29-31^. The coverage of clustered ORF sequences was determined by mapping short reads against the assembly genomes of each sample using bbmap.sh (v37.68) to provide coverage information. Then, pileup.sh was used to convert the bbmap coverage data to reads per kilobase per million (RPKM) as previously described ^31^. The horizontal gene transfer (HGT) events among rMAGs were identified using MetaCHIP (v1.10.13) ^32^.

# Supplementary Text 3. Data analysis and statistics

The α-diversity (Shannon index and richness) and adonis test for microbiota and ARGs were performed using the "vegan" (v2.6-4) package in R (v4.2.2). Principal Coordinates Analysis (PCoA) and adonis2 test were performed to distinguish microbial composition, ARGs and functional profiles in different groups using Bray-Curtis dissimilarity using the "vegan" (v2.6-4) package, respectively. Differential microbial composition and functional traits between groups were identified using the "edgeR" (v3.40) package. All figures depicting taxonomic and functional visualizations were created in R. Phylogenetic tree was visualized using the iTOL tool (v.6.8, https://itol.embl.de/) ^33^. Assembled genomes including rMAGs and contig that exhibited significant changes were selected based on their relative abundance as determined by coverM and MetaWrap. Relative abundance of a contig was calculated as contig coverage divided by sample size (in gb). In addition, functional differences were selected based on the normalized abundance of KO pathway (transcripts per million; TPM) as provided by eggNOG-mapper and bbmap. Differences in taxonomic and functional compositions were assessed using edgeR, and biomarker identification across groups was conducted with LEfSe analysis. The *p* values were adjusted using the false discovery rate (FDR), with statistical significance defined at a 95% confidence interval (*p* < 0.05), unless otherwise noted. Results were presented as the mean ± standard deviation (SD) in all bar plots. Box plots displayed the median as the center line, with the first and third quartiles as the box limits.


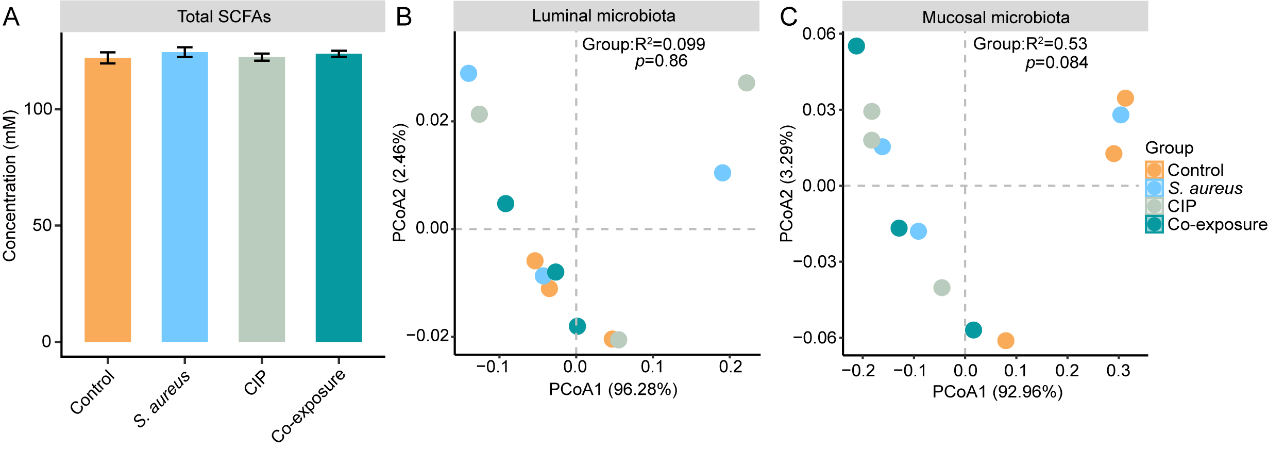


Fig. S1 The microbial compositions and metabolic activities in different groups after the stabilization period. **(A)** Total production of short chain fatty acids (SCFAs) including isobutyrate, isovalerate, isocaproate, acetate, propionate, and butyrate in different groups after the stabilization period. The Principal Coordinates Analysis (PCoA) analysis of the **(B)** luminal and **(C)** mucosal microbial compositions in different groups after the stabilization period based on the Bray–Curtis dissimilarity.


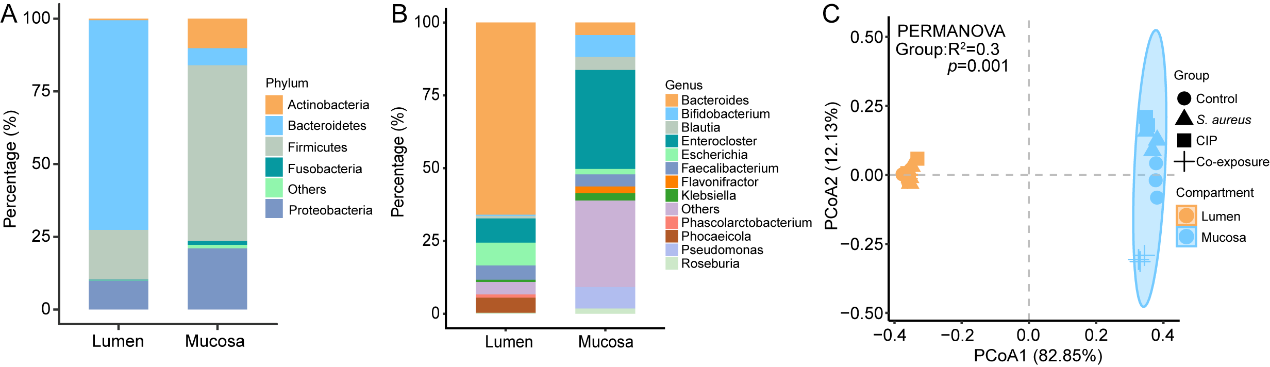


Fig. S2 The compositions of luminal and mucosal microbiota in all groups after the stabilization period. **(A)** The average relative abundance of phyla in luminal and mucosal microbiota after the stabilization period. **(B)** The average relative abundance of genera in luminal and mucosal microbiota after the stabilization period. **(C)** The Principal Coordinates Analysis (PCoA) analysis of the luminal and mucosal microbial compositions in different groups after the stabilization period based on the Bray–Curtis dissimilarity.


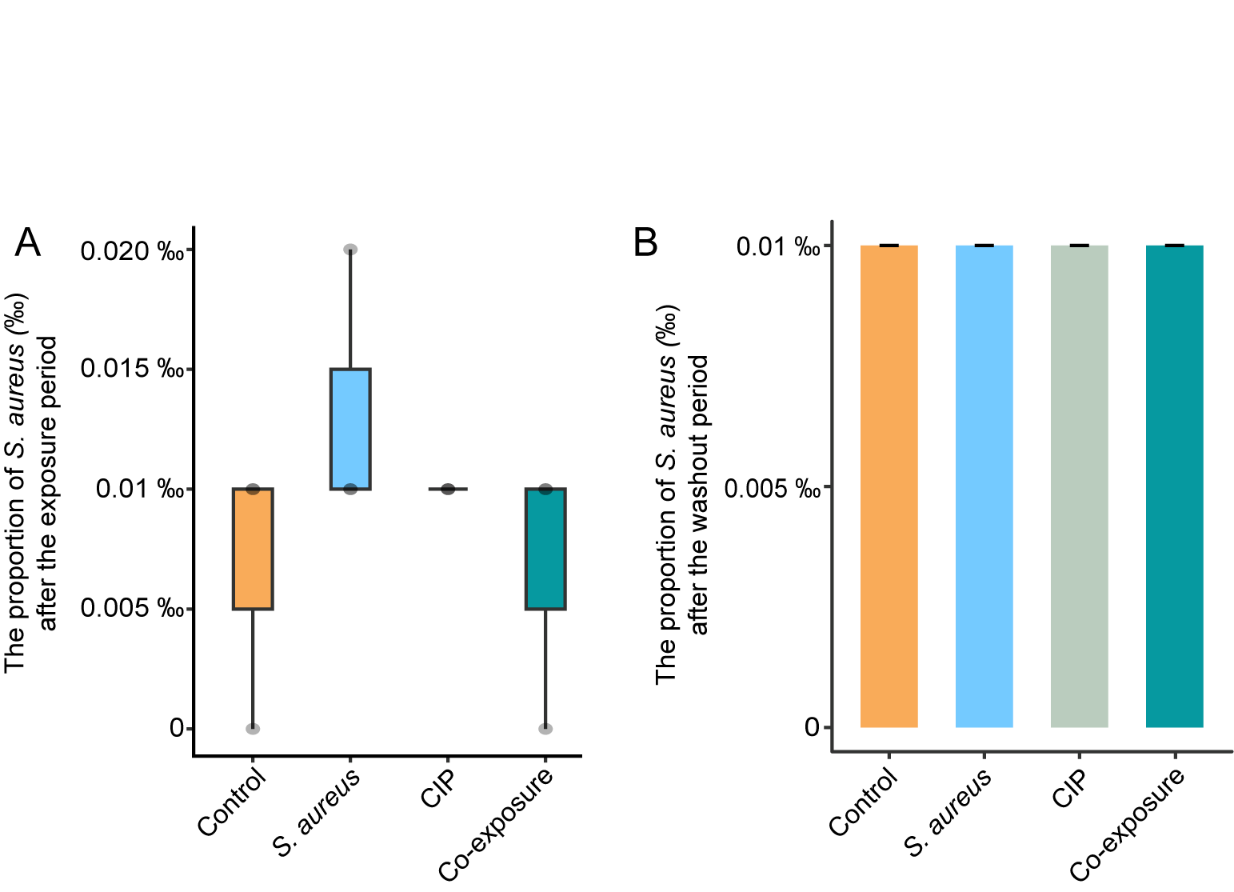


Fig. S3 The relative abundance of *S. aureus* in luminal microbiota at short-read level after the (A) exposure and (B) washout period, respectively.


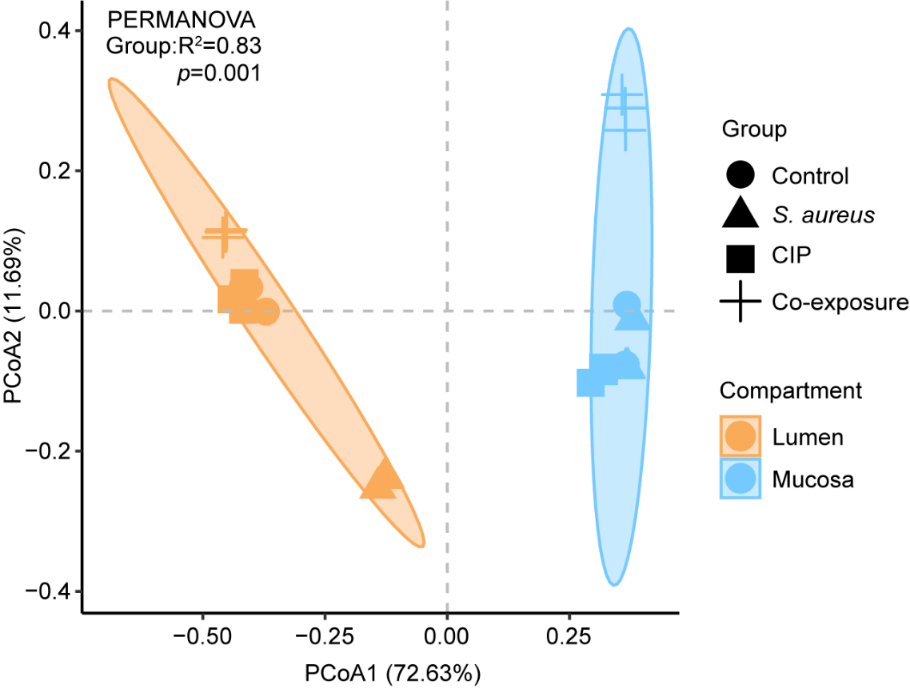


Fig. S4 The Principal Coordinates Analysis (PCoA) analysis of the luminal and mucosal microbial compositions in different groups after the exposure period based on the Bray–Curtis dissimilarity.


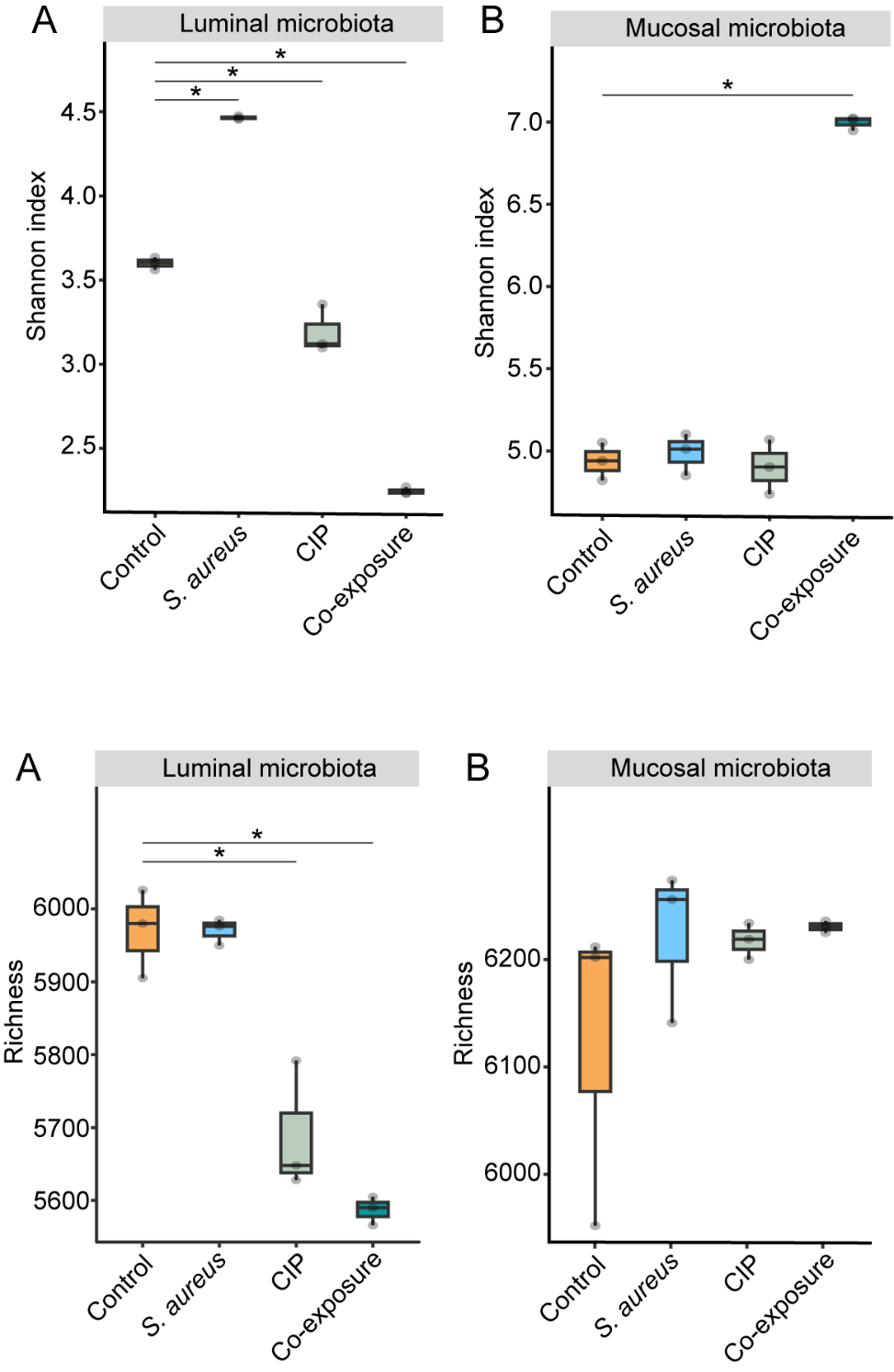


Fig. S5 The species richness of (A) luminal and (B) mucosal microbiota in different groups after the exposure period. * denotes *p* < 0.05 based on the Kruskal-Wallis H-test between the corresponding treatment group and control group.


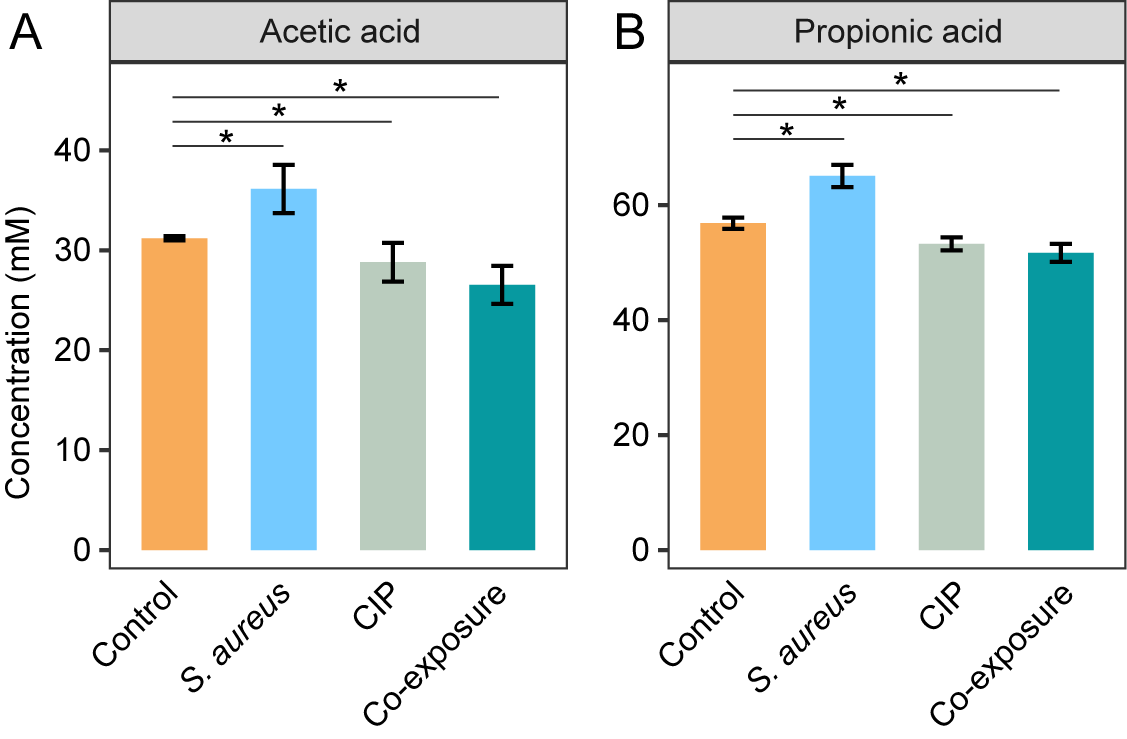


Fig. S6 Production of (A) acetate and (B) propionate in different groups after the exposure period. * denotes *p* < 0.05 based on the Kruskal-Wallis H-test between the corresponding treatment group and control group.


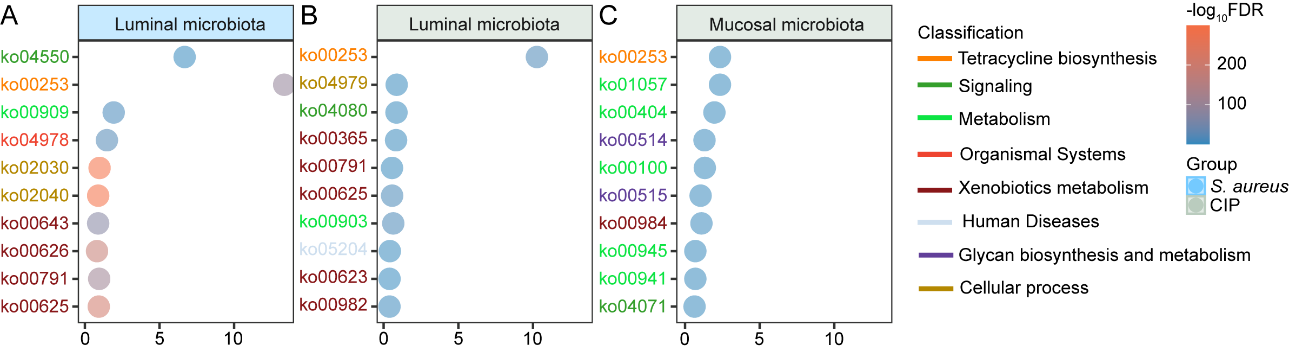


Fig. S7 The significantly increased top 10 KO pathways in (A) luminal microbiota of *S. aureus* group, and (B) luminal and (C) mucosal microbiota of CIP group based on the fold change compared to the control group after the exposure period.


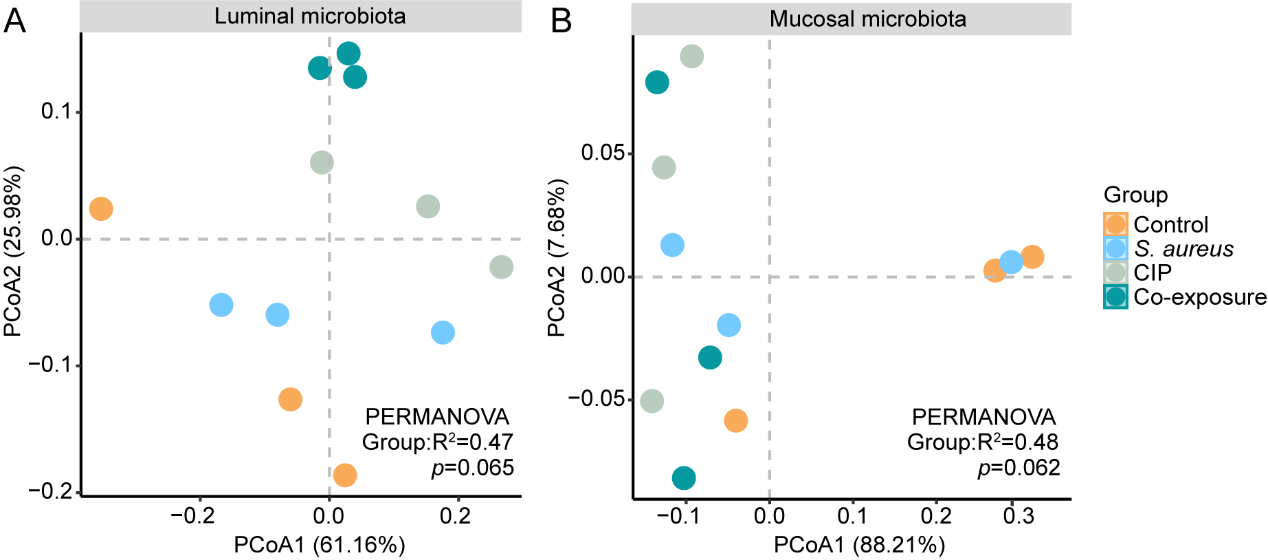


Fig. S8 The Principal Coordinates Analysis (PCoA) analysis of the ARGs composition in (A) luminal and (B) mucosal microbiota in different groups after the exposure period based on the Bray–Curtis dissimilarity.


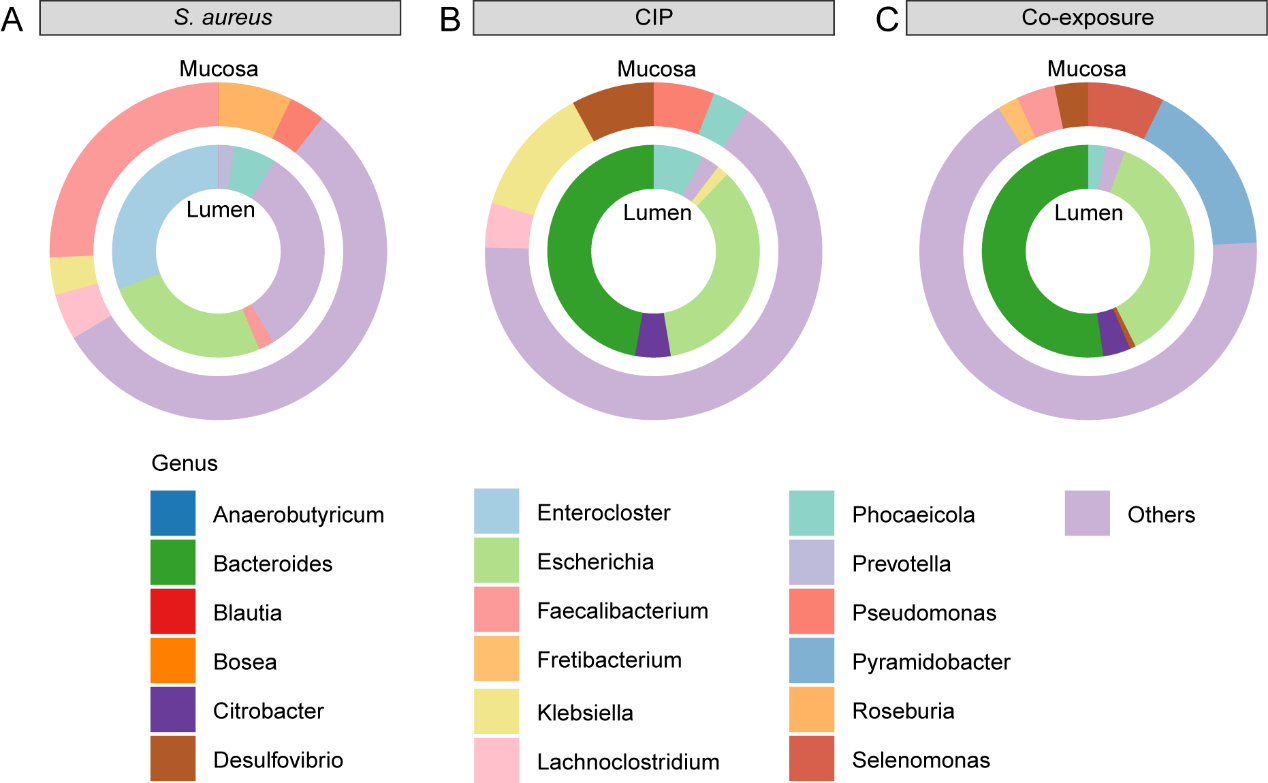


Fig. S9 The average relative abundance of genera in the significantly increased ARG-carrying contigs (ACCs) of (A) *S. aureus*, (B) CIP and (C) co-exposure groups after the exposure period.


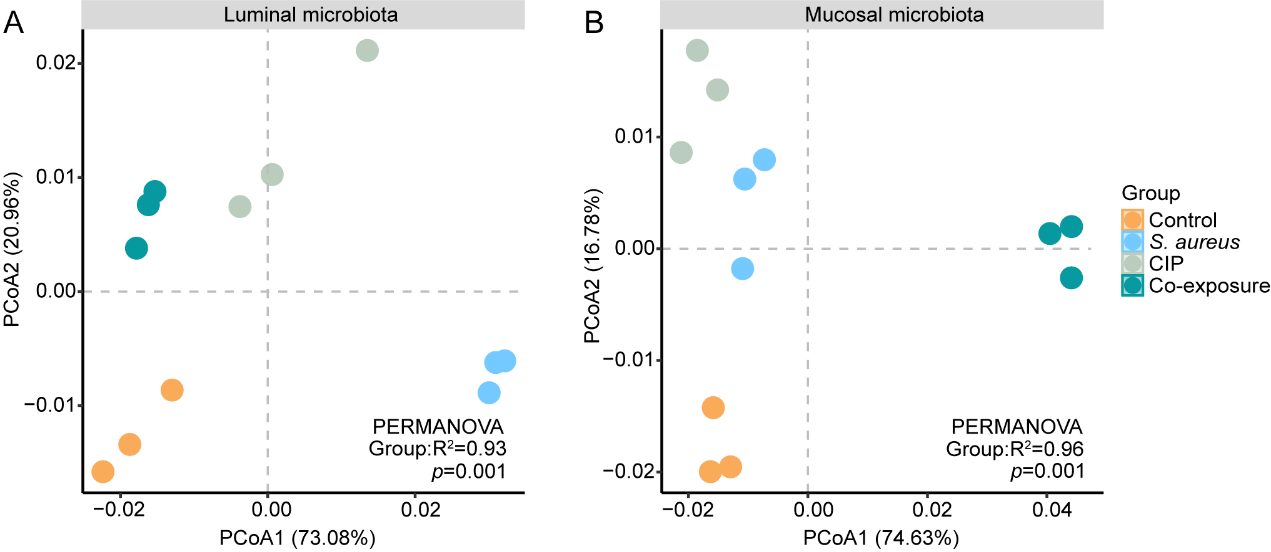


Fig. S10 The Principal Coordinates Analysis (PCoA) analysis of the composition of KEGG Orthology (KO) pathways in contigs of (A) luminal and (B) mucosal microbiota in different groups after the exposure period based on the Bray–Curtis dissimilarity.


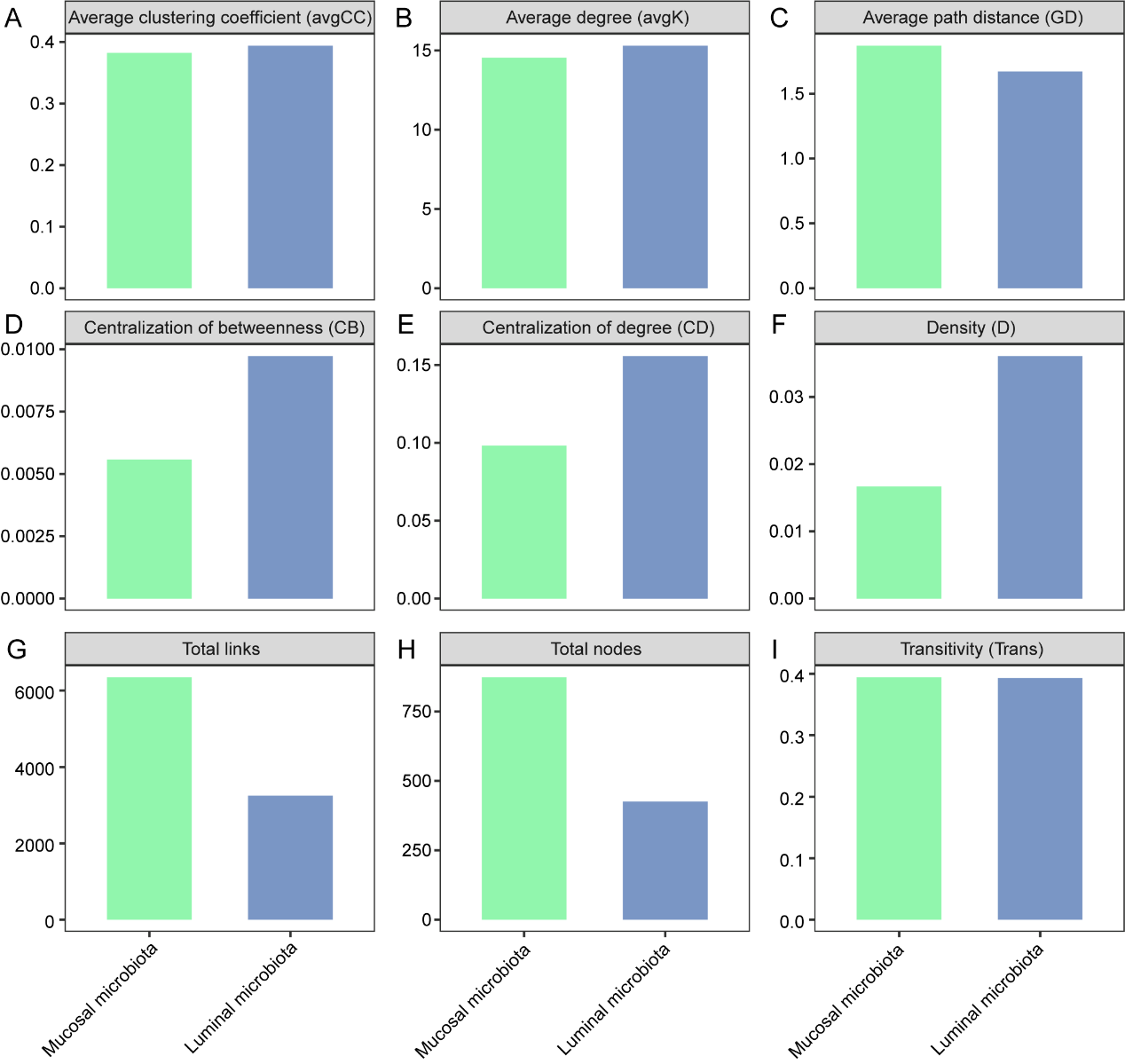


Fig. S11 Topological parameters in the microbial networks of luminal and mucosal microbiota **at contig level after the exposure period**, respectively. The **(A)** average clustering coefficient (avgCC), **(B)** average degree (avgK), **(C)** average path distance (GD), **(D)** centralization of betweenness (CB), **(E)** centralization of degree (CD), **(F)** density (D), **(G)** total nodes, **(H)** total links and **(I)** transitivity (Trans) in the microbial networks of luminal and mucosal microbiota after the exposure period, respectively.


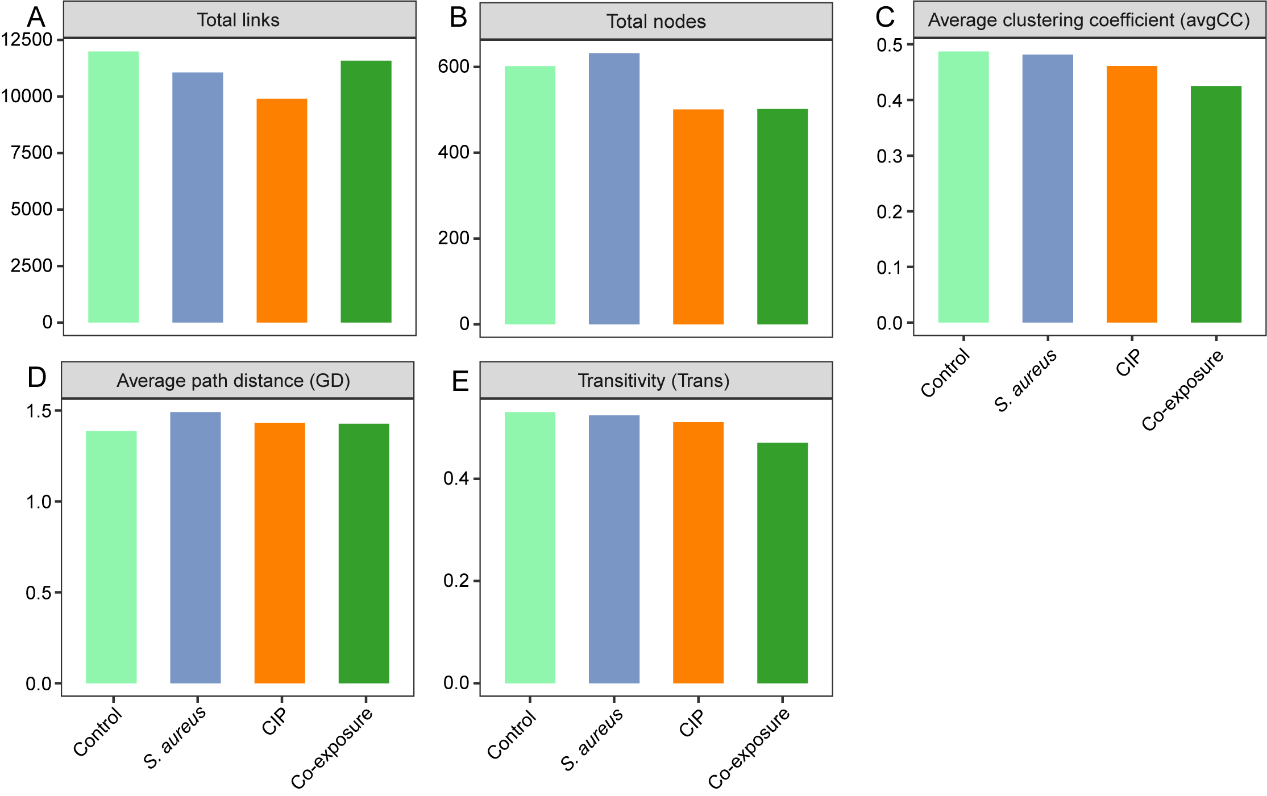


Fig. S12 Topological parameters in the microbial networks of different groups after the exposure period, respectively. The (A) total nodes, (B) total links, (C) average clustering coefficient (avgCC), (D) average path distance (GD) and (E) transitivity (Trans) in the microbial networks of different groups after the exposure period, respectively.


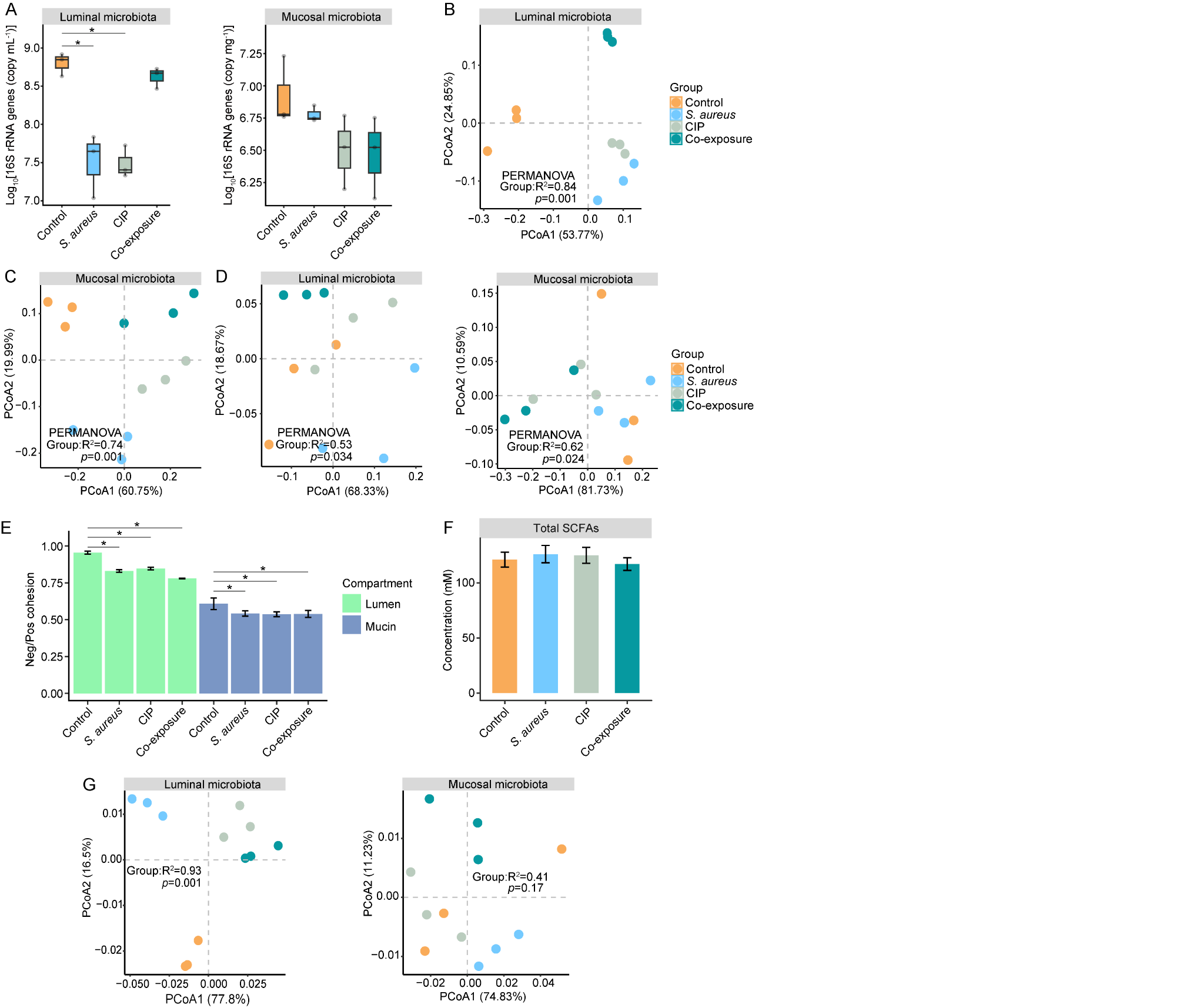


Fig. S13 The microbial compositions, antibiotic resistance profiles, and fermentation activities of gut microbiota in different groups after the washout period. **(A)** The absolute abundance of 16S rRNA genes in the luminal and mucosal microbiota of different groups after the washout period, respectively. * denotes *p* < 0.05 based on the Kruskal-Wallis H-test between the corresponding treatment group and control group. **(B-D)** The Principal Coordinates Analysis (PCoA) analysis of the (B) luminal and (C) mucosal microbial compositions and (D) ARGs compositions in luminal and mucosal microbiota in different groups after the washout period based on the Bray–Curtis dissimilarity. **(E)** The ratio of negative:positive cohesion in gut microbiota of different compartments and groups after the washout period, respectively. * denotes *p* < 0.05 based on the Kruskal-Wallis H-test between the corresponding treatment group and control group. **(F)** Total production of short chain fatty acids (SCFAs) including isobutyrate, isovalerate, isocaproate, acetate, propionate, and butyrate in different groups after the washout period. * denotes *p* < 0.05 based on the Kruskal-Wallis H-test between the corresponding treatment group and control group.

**Supplementary Table 1.** The information of the close-related complete genome with the isolated *S. aureus* from NCBI (<https://www.ncbi.nlm.nih.gov/pathogens>)

| AMR genotypes | Strain | Isolation source | Isolation type | Location | Level | Organism group |
| --- | --- | --- | --- | --- | --- | --- |
| blaI, blaPC1, blaR1, fosB, glpT_A100V, mepA, tet(38) | *014S_SA* | Human skin and nose ^34^ | clinical | Samoa | Complete Genome | *S. aureus* |
| blaI, blaPC1, blaR1, fosB, glpT_A100V, mepA, tet(38) | *ST20130942* | 88-year-old man presenting knee prosthesis infection | clinical | France |  |  |
| blaI, blaPC1, blaR1, fosB, glpT_A100V, mepA, tet(38) | *ST20130943* | 88-year-old man presenting knee prosthesis infection | clinical | France |  |  |
| blaI, blaPC1, blaR1, fosB, glpT_A100V, mepA, tet(38) | *UP_883* |  | clinical |  |  |  |
| blaI, blaPC1, blaR1, fosB, glpT_A100V, mepA, tet(38) | *UP_274* |  | clinical |  |  |  |
| blaI, blaPC1, blaR1, fosB, glpT_A100V, mepA, tet(38) | *NAS_OP_163* |  | clinical |  |  |  |
| blaI, blaPC1, blaR1, fosB, glpT_A100V, mepA, tet(38) | *NAS_AN_265* |  | clinical |  |  |  |
| blaI, blaPC1, blaR1, fosB, glpT_A100V, mepA, parC_S80F, tet(38) | *SA12-SX* | purulent fluid | clinical | China |  |  |
| blaI, blaPC1, blaR1, dfrG, fosB, glpT_A100V, mepA, parC_S80F, tet(38) | *SA08-SX* | secretion | clinical | China |  |  |

**Supplementary Table 2.** The proportion of life-history strategies in each group and the proportion of level 2 classification in them

| Group | Compartment | A | Competition | Motility and chemotaxis | Substrate utilization | S | Cell communication | Cell homeostasis maintenance | Y | Biosynthesis | Cell organization and biogenesis | Central metabolism |
| --- | --- | --- | --- | --- | --- | --- | --- | --- | --- | --- | --- | --- |
| *S. aureus* | Lumen | 40.93 ± 0.02 | 1.58 ± 0.03 | 4.39 ± 0.02 | 94.03 ± 0.05 | 31.08  ± 0.10 | 36.67 ± 0.04 | 63.33 ± 0.04 | 26.04  ± 0.12 | 38 ± 0.14 | 38.78 ± 0.24 | 23.22 ± 0.11 |
|  | Mucosa | 56.74 ± 0.01 | 2.77 ± 0.05 | 0.00 | 97.23 ± 0.05 | 16.44  ± 0.03 | 24.99 ± 0.57 | 75.01 ± 0.57 | 12.32  ± 0.18 | 13.64 ± 0.05 | 17.25 ± 0.17 | 69.11 ± 0.16 |
| CIP | Lumen | 27.11 ± 0.10 | 0.00 | 0.00 | 100.00 | 44.6  ± 0.35 | 13.05 ± 0.22 | 86.95 ± 0.22 | 7.8  ± 0.26 | 47.62 ± 0.32 | 8.76 ± 0.10 | 43.61 ± 0.23 |
|  | Mucosa | 63.41 ± 0.15 | 1.46 ± 0.03 | 0.00 | 98.54 ± 0.03 | 11.14  ± 0.15 | 40.57 ± 0.25 | 59.43 ± 0.25 | 11.97  ± 0.18 | 10.71 ± 0.07 | 0.84 ± 0.03 | 88.45 ± 0.04 |
| co-exposure | Lumen | 54.2 ± 0.03 | 5.35 ± 0.01 | 0.00 | 94.65 ± 0.01 | 16.27  ± 0.04 | 16.07 ± 0.09 | 83.93 ± 0.09 | 28.62  ± 0.20 | 30.54 ± 0.03 | 33.05 ± 0.02 | 36.41 ± 0.04 |
|  | Mucosa | 51.72 ± 0.12 | 4.82 ± 0.05 | 0.00 | 95.18 ± 0.05 | 22.87  ± 0.09 | 39.21 ± 0.12 | 60.79 ± 0.12 | 29.4  ± 0.09 | 32.3 ± 0.30 | 49.79 ± 0.27 | 17.91 ± 0.05 |

**Supplementary Table 3.** Classification of life-history strategies based on KO pathways ^11, 12^

| Level 1  (life-history strategy) | Level 2 | Level 3  (ko pathway) |
| --- | --- | --- |
| A | Substrate utilization | Glycine, serine and threonine metabolism |
| A | Motility and chemotaxis | Flagellar assembly |
| A | Motility and chemotaxis | Bacterial chemotaxis |
| A | Substrate utilization | Cysteine and methionine metabolism |
| A | Substrate utilization | Arginine and proline metabolism |
| A | Substrate utilization | Phenylalanine metabolism |
| A | Substrate utilization | Tyrosine metabolism |
| A | Substrate utilization | Lysine degradation |
| A | Substrate utilization | Tryptophan metabolism |
| A | Competition | Prodigiosin biosynthesis |
| A | Competition | Phenazine biosynthesis |
| A | Competition | Glucosinolate biosynthesis |
| A | Substrate utilization | Pyruvate metabolism |
| A | Substrate utilization | Fructose and mannose metabolism |
| A | Substrate utilization | Pentose phosphate pathway |
| A | Substrate utilization | Butanoate metabolism |
| A | Substrate utilization | Ascorbate and aldarate metabolism |
| A | Substrate utilization | Inositol phosphate metabolism |
| A | Substrate utilization | Glycerolipid metabolism |
| A | Substrate utilization | Fatty acid degradation |
| A | Substrate utilization | Porphyrin metabolism |
| A | Substrate utilization | Retinol metabolism |
| A | Substrate utilization | Selenocompound metabolism |
| A | Substrate utilization | Limonene degradation |
| A | Substrate utilization | Purine metabolism |
| A | Substrate utilization | Pyrimidine metabolism |
| A | Substrate utilization | Benzoate degradation |
| A | Substrate utilization | Aminobenzoate degradation |
| A | Substrate utilization | Chloroalkane and chloroalkene degradation |
| A | Substrate utilization | Nitrotoluene degradation |
| A | Substrate utilization | Naphthalene degradation |
| A | Substrate utilization | Xylene degradation |
| A | Substrate utilization | Dioxin degradation |
| A | Substrate utilization | Drug metabolism - cytochrome P450 |
| A | Substrate utilization | Metabolism of xenobiotics by cytochrome P450 |
| A | Substrate utilization | Styrene degradation |
| A | Substrate utilization | Toluene degradation |
| A | Substrate utilization | Chlorocyclohexane and chlorobenzene degradation |
| A | Substrate utilization | Caprolactam degradation |
| A | Substrate utilization | Atrazine degradation |
| A | Substrate utilization | Furfural degradation |
| A | Substrate utilization | Mineral absorption |
| A | Substrate utilization | Cholesterol metabolism |
| A | Substrate utilization | Alanine, aspartate and glutamate metabolism |
| A | Competition | Carbapenem biosynthesis |
| A | Competition | Flavone and flavonol biosynthesis |
| A | Competition | Flavonoid biosynthesis |
| A | Competition | Stilbenoid, diarylheptanoid and gingerol biosynthesis |
| A | Substrate utilization | Citrate cycle (TCA cycle) |
| A | Substrate utilization | Glyoxylate and dicarboxylate metabolism |
| A | Substrate utilization | One carbon pool by folate |
| A | Substrate utilization | Biotin metabolism |
| A | Substrate utilization | Riboflavin metabolism |
| A | Substrate utilization | Phosphonate and phosphinate metabolism |
| A | Substrate utilization | beta-Alanine metabolism |
| A | Substrate utilization | Drug metabolism - other enzymes |
| A | Substrate utilization | Glutathione metabolism |
| A | Substrate utilization | Carbohydrate digestion and absorption |
| A | Substrate utilization | Valine, leucine and isoleucine degradation |
| A | Competition | Staurosporine biosynthesis |
| A | Substrate utilization | Propanoate metabolism |
| A | Substrate utilization | Pentose and glucuronate interconversions |
| A | Substrate utilization | Ether lipid metabolism |
| A | Substrate utilization | alpha-Linolenic acid metabolism |
| A | Substrate utilization | Linoleic acid metabolism |
| A | Substrate utilization | Arachidonic acid metabolism |
| A | Substrate utilization | Fluorobenzoate degradation |
| A | Substrate utilization | Ethylbenzene degradation |
| A | Substrate utilization | Steroid degradation |
| A | Competition | Monobactam biosynthesis |
| A | Competition | Neomycin, kanamycin and gentamicin biosynthesis |
| A | Competition | Streptomycin biosynthesis |
| A | Competition | Acarbose and validamycin biosynthesis |
| A | Substrate utilization | C5-Branched dibasic acid metabolism |
| A | Substrate utilization | Glycolysis / Gluconeogenesis |
| A | Substrate utilization | Amino sugar and nucleotide sugar metabolism |
| A | Substrate utilization | Galactose metabolism |
| A | Substrate utilization | Sphingolipid metabolism |
| A | Substrate utilization | Vitamin B6 metabolism |
| A | Substrate utilization | Thiamine metabolism |
| A | Substrate utilization | Peroxisome |
| A | Substrate utilization | Histidine metabolism |
| A | Competition | Novobiocin biosynthesis |
| A | Competition | Tropane, piperidine and pyridine alkaloid biosynthesis |
| A | Competition | Isoquinoline alkaloid biosynthesis |
| A | Competition | Penicillin and cephalosporin biosynthesis |
| A | Competition | Betalain biosynthesis |
| A | Substrate utilization | Glycerophospholipid metabolism |
| A | Substrate utilization | Nicotinate and nicotinamide metabolism |
| A | Substrate utilization | Lipoic acid metabolism |
| A | Substrate utilization | Cyanoamino acid metabolism |
| A | Substrate utilization | Taurine and hypotaurine metabolism |
| A | Substrate utilization | Autophagy - yeast |
| A | Substrate utilization | Polycyclic aromatic hydrocarbon degradation |
| S | Cell communication | Signaling pathways regulating pluripotency of stem cells |
| S | Cell communication | Quorum sensing |
| S | Cell communication | Biofilm formation - Pseudomonas aeruginosa |
| S | Cell homeostasis maintenance | Platinum drug resistance |
| S | Cell homeostasis maintenance | Mismatch repair |
| S | Cell communication | Two-component system |
| S | Cell communication | Neuroactive ligand-receptor interaction |
| S | Cell homeostasis maintenance | Peptidoglycan biosynthesis |
| S | Cell homeostasis maintenance | N-Glycan biosynthesis |
| S | Cell homeostasis maintenance | ABC transporters |
| S | Cell homeostasis maintenance | Bacterial secretion system |
| S | Cell homeostasis maintenance | Phosphotransferase system (PTS) |
| S | Cell homeostasis maintenance | Proximal tubule bicarbonate reclamation |
| S | Cell communication | Prolactin signaling pathway |
| S | Cell homeostasis maintenance | Homologous recombination |
| S | Cell homeostasis maintenance | DNA replication |
| S | Cell communication | Biofilm formation - Vibrio cholerae |
| S | Cell homeostasis maintenance | Cationic antimicrobial peptide (CAMP) resistance |
| S | Cell homeostasis maintenance | beta-Lactam resistance |
| S | Cell communication | FoxO signaling pathway |
| S | Cell communication | MAPK signaling pathway - yeast |
| S | Cell communication | Sphingolipid signaling pathway |
| S | Cell communication | p53 signaling pathway |
| S | Cell homeostasis maintenance | Various types of N-glycan biosynthesis |
| S | Cell homeostasis maintenance | Mannose type O-glycan biosynthesis |
| S | Cell homeostasis maintenance | Other types of O-glycan biosynthesis |
| S | Cell communication | RIG-I-like receptor signaling pathway |
| S | Cell homeostasis maintenance | Other glycan degradation |
| S | Cell communication | Biofilm formation - Escherichia coli |
| S | Cell homeostasis maintenance | Base excision repair |
| S | Cell homeostasis maintenance | Nucleotide excision repair |
| S | Cell homeostasis maintenance | Lipopolysaccharide biosynthesis |
| S | Cell communication | MAPK signaling pathway - fly |
| S | Cell homeostasis maintenance | Thyroid hormone synthesis |
| S | Cell homeostasis maintenance | Antifolate resistance |
| S | Cell communication | Glucagon signaling pathway |
| S | Cell communication | HIF-1 signaling pathway |
| S | Cell communication | MAPK signaling pathway - plant |
| S | Cell communication | AMPK signaling pathway |
| S | Cell homeostasis maintenance | Glycosphingolipid biosynthesis - globo and isoglobo series |
| S | Cell homeostasis maintenance | Glycosaminoglycan degradation |
| S | Cell communication | Insulin signaling pathway |
| S | Cell homeostasis maintenance | Thermogenesis |
| S | Cell communication | Phosphatidylinositol signaling system |
| S | Cell communication | Phospholipase D signaling pathway |
| S | Cell communication | Adipocytokine signaling pathway |
| Y | Biosynthesis | Phenylalanine, tyrosine and tryptophan biosynthesis |
| Y | Biosynthesis | Lysine biosynthesis |
| Y | Biosynthesis | Arginine biosynthesis |
| Y | Biosynthesis | Valine, leucine and isoleucine biosynthesis |
| Y | Central metabolism | Methane metabolism |
| Y | Central metabolism | Carbon fixation in photosynthetic organisms |
| Y | Central metabolism | Nitrogen metabolism |
| Y | Central metabolism | Photosynthesis |
| Y | Central metabolism | Photosynthesis - antenna proteins |
| Y | Cell organization and biogenesis | Sulfur relay system |
| Y | Cell organization and biogenesis | Proteasome |
| Y | Biosynthesis | Biosynthesis of unsaturated fatty acids |
| Y | Biosynthesis | Pantothenate and CoA biosynthesis |
| Y | Biosynthesis | Ubiquinone and other terpenoid-quinone biosynthesis |
| Y | Biosynthesis | Terpenoid backbone biosynthesis |
| Y | Biosynthesis | Biosynthesis of ansamycins |
| Y | Biosynthesis | Biosynthesis of siderophore group nonribosomal peptides |
| Y | Biosynthesis | Insect hormone biosynthesis |
| Y | Biosynthesis | Tetracycline biosynthesis |
| Y | Biosynthesis | Sesquiterpenoid and triterpenoid biosynthesis |
| Y | Cell organization and biogenesis | RNA polymerase |
| Y | Cell organization and biogenesis | Basal transcription factors |
| Y | Cell organization and biogenesis | Ribosome |
| Y | Cell organization and biogenesis | Aminoacyl-tRNA biosynthesis |
| Y | Cell organization and biogenesis | Cell cycle - Caulobacter |
| Y | Cell organization and biogenesis | Apoptosis |
| Y | Central metabolism | Carbon fixation pathways in prokaryotes |
| Y | Cell organization and biogenesis | RNA degradation |
| Y | Biosynthesis | Folate biosynthesis |
| Y | Cell organization and biogenesis | Nucleocytoplasmic transport |
| Y | Cell organization and biogenesis | Osteoclast differentiation |
| Y | Central metabolism | Sulfur metabolism |
| Y | Biosynthesis | Steroid hormone biosynthesis |
| Y | Biosynthesis | Steroid biosynthesis |
| Y | Biosynthesis | Biosynthesis of type II polyketide products |
| Y | Central metabolism | Oxidative phosphorylation |
| Y | Central metabolism | Protein export |
| Y | Biosynthesis | Zeatin biosynthesis |
| Y | Biosynthesis | Polyketide sugar unit biosynthesis |
| Y | Cell organization and biogenesis | Necroptosis |
| Y | Cell organization and biogenesis | Meiosis - yeast |
| Y | Central metabolism | Protein processing in endoplasmic reticulum |
| Y | Biosynthesis | Biosynthesis of vancomycin group antibiotics |
| Y | Cell organization and biogenesis | Ribosome biogenesis in eukaryotes |
| Y | Cell organization and biogenesis | Nonribosomal peptide structures |
| Y | Cell organization and biogenesis | Ferroptosis |
| U | - | Pertussis |
| U | - | Tuberculosis |
| U | - | Legionellosis |
| U | - | Bacterial invasion of epithelial cells |
| U | - | Amoebiasis |
| U | - | African trypanosomiasis |
| U | - | Influenza A |
| U | - | Human T-cell leukemia virus one infection |
| U | - | MicroRNAs in cancer |
| U | - | Bladder cancer |
| U | - | Renal cell carcinoma |
| U | - | Cardiac muscle contraction |
| U | - | Non-alcoholic fatty liver disease |
| U | - | Cushing syndrome |
| U | - | Alzheimer disease |
| U | - | Parkinson disease |
| U | - | Prion disease |
| U | - | Epithelial cell signaling in Helicobacter pylori infection |
| U | - | Staphylococcus aureus infection |
| U | - | Toxoplasmosis |
| U | - | Hepatitis B |
| U | - | Kaposi sarcoma-associated herpesvirus infection |
| U | - | Herpes simplex virus one infection |
| U | - | Longevity regulating pathway |
| U | - | Colorectal cancer |
| U | - | Small cell lung cancer |
| U | - | Viral myocarditis |
| U | - | GABAergic synapse |
| U | - | Glutamatergic synapse |
| U | - | Amyotrophic lateral sclerosis |
| U | - | Longevity regulating pathway - worm |
| U | - | Chemical carcinogenesis - DNA adducts |
| U | - | Hepatocellular carcinoma |
| U | - | Insulin resistance |
| U | - | Rheumatoid arthritis |
| U | - | Vibrio cholerae infection |
| U | - | Huntington disease |
| U | - | Salmonella infection |
| U | - | Shigellosis |
| U | - | Longevity regulating pathway - multiple species |
| U | - | Central carbon metabolism in cancer |
| U | - | Choline metabolism in cancer |
| U | - | Type II diabetes mellitus |
| U | - | Type I diabetes mellitus |
| U | - | Human papillomavirus infection |
| U | - | Viral carcinogenesis |
| U | - | Proteoglycans in cancer |

**Supplementary Table 4.** Topological properties of the networks in different compartments and groups after exposure period.

|  | Network Index | Luminal microbiota | Mucosal microbiota | Control | *S. aureus* | Ciprofloxacin  (CIP) | Co-exposure |
| --- | --- | --- | --- | --- | --- | --- | --- |
| Empirical network | Total nodes | 255 | 442 | 328 | 345 | 310 | 354 |
|  | Total links | 3250 | 6347 | 11988 | 11062 | 9894 | 11581 |
|  | Average clustering coefficient (avgCC) | 0.39 | 0.38 | 0.49 | 0.48 | 0.46 | 0.43 |
|  | Transitivity (Trans) | 0.39 | 0.39 | 0.53 | 0.52 | 0.51 | 0.47 |
|  | Modularity | 0.26 | 0.39 | 0.1 | 0.09 | 0.1 | 0.09 |
|  | Centralization of eigenvector centrality (CE) | 0.83 | 0.88 | 0.78 | 0.79 | 0.77 | 0.73 |
|  | Geodesic efficiency (E) | 0.23 | 0.16 | 0.24 | 0.23 | 0.3 | 0.39 |
|  | Centralization of degree (CD) | 0.16 | 0.1 | 0.23 | 0.23 | 0.26 | 0.25 |
|  | Centralization of betweenness (CB) | 0.01 | 0.01 | 0.01 | 0.01 | 0.01 | 0.01 |
|  | Centralization of stress centrality (CS) | 0.15 | 0.15 | 0.25 | 0.21 | 0.3 | 0.34 |
| Random networks | avgCC | 0.32 ± 0.01 | 0.23 ± 0.01 | 0.47 ± 0.01 | 0.49 ± 0.01 | 0.46 ± 0.01 | 0.42 ± 0.01 |
|  | Trans | 0.3 ± 0 | 0.22 ± 0 | 0.51 ± 0 | 0.51 ± 0 | 0.5 ± 0 | 0.46 ± 0 |
|  | Modularity | 0.12 ± 0 | 0.12 ± 0 | 0.07 ± 0 | 0.06 ± 0 | 0.07 ± 0 | 0.07 ± 0 |
|  | CE | 0.81 ± 0 | 0.85 ± 0 | 0.77 ± 0 | 0.79 ± 0 | 0.76 ± 0 | 0.72 ± 0 |
|  | E | 0.26 ± 0 | 0.17 ± 0 | 0.250 ± 0 | 0.24 ± 0 | 0.31 ± 0 | 0.4 ± 0 |
|  | CD | 0.16 ± 0 | 0.1 ± 0 | 0.23 ± 0 | 0.23 ± 0 | 0.26 ± 0 | 0.25 ± 0 |
|  | CB | 0.01 ± 0 | 0.01 ± 0 | 0 ± 0 | 0.01 ± 0 | 0.01 ± 0 | 0.01 ± 0 |
|  | CS | 0.13 ± 0.02 | 0.08 ± 0.01 | 0.09 ± 0.01 | 0.11 ± 0.01 | 0.14 ± 0.02 | 0.17 ± 0.02 |

**Supplementary Table 5**. Potential HGT events of the significantly increased biomarkers in different groups.

| Group | Compartment | Classification | KO name | Description | HGT gene number |
| --- | --- | --- | --- | --- | --- |
| *S. aureus* | Lumen | Donor | K02003 | ABC.CD.A; putative ABC transport system ATP-binding protein | 1 |
| *S. aureus* | Lumen | Donor | K02013 | ABC.FEV.A; iron complex transport system ATP-binding protein [EC:7.2.2.-] | 1 |
| *S. aureus* | Lumen | Donor | K02015 | ABC.FEV.P; iron complex transport system permease protein | 1 |
| *S. aureus* | Lumen | Donor | K02033 | ABC.PE.P; peptide/nickel transport system permease protein | 1 |
| *S. aureus* | Lumen | Donor | K06147 | ABCB-BAC; ATP-binding cassette, subfamily B, bacterial | 2 |
| *S. aureus* | Lumen | Donor | K01681 | ACO, acnA; aconitate hydratase [EC:4.2.1.3] | 1 |
| *S. aureus* | Lumen | Donor | K00073 | allD; ureidoglycolate dehydrogenase (NAD+) [EC:1.1.1.350] | 1 |
| *S. aureus* | Lumen | Donor | K03077 | araD, ulaF, sgaE, sgbE; L-ribulose-5-phosphate 4-epimerase [EC:5.1.3.4] | 1 |
| *S. aureus* | Lumen | Donor | K00926 | arcC; carbamate kinase [EC:2.7.2.2] | 1 |
| *S. aureus* | Lumen | Donor | K03564 | BCP, PRXQ, DOT5; thioredoxin-dependent peroxiredoxin [EC:1.11.1.24] | 1 |
| *S. aureus* | Lumen | Donor | K00108 | betA, CHDH; choline dehydrogenase [EC:1.1.99.1] | 1 |
| *S. aureus* | Lumen | Donor | K00130 | betB, gbsA; betaine-aldehyde dehydrogenase [EC:1.2.1.8] | 1 |
| *S. aureus* | Lumen | Donor | K19271 | catA; chloramphenicol O-acetyltransferase type A [EC:2.3.1.28] | 1 |
| *S. aureus* | Lumen | Donor | K02190 | cbiK; sirohydrochlorin cobaltochelatase [EC:4.99.1.3] | 1 |
| *S. aureus* | Lumen | Donor | K15256 | cmoA; tRNA (cmo5U34)-methyltransferase [EC:2.1.1.-] | 1 |
| *S. aureus* | Lumen | Donor | K01715 | crt; enoyl-CoA hydratase [EC:4.2.1.17] | 1 |
| *S. aureus* | Lumen | Donor | K00525 | E1.17.4.1A, nrdA, nrdE; ribonucleoside-diphosphate reductase alpha chain [EC:1.17.4.1] | 1 |
| *S. aureus* | Lumen | Donor | K00526 | E1.17.4.1B, nrdB, nrdF; ribonucleoside-diphosphate reductase beta chain [EC:1.17.4.1] | 1 |
| *S. aureus* | Lumen | Donor | K01678 | E4.2.1.2AB, fumB; fumarate hydratase subunit beta [EC:4.2.1.2] | 1 |
| *S. aureus* | Lumen | Donor | K01848 | E5.4.99.2A, mcmA1; methylmalonyl-CoA mutase, N-terminal domain [EC:5.4.99.2] | 1 |
| *S. aureus* | Lumen | Donor | K01849 | E5.4.99.2B, mcmA2; methylmalonyl-CoA mutase, C-terminal domain [EC:5.4.99.2] | 1 |
| *S. aureus* | Lumen | Donor | K01625 | eda; 2-dehydro-3-deoxyphosphogluconate aldolase / (4S)-4-hydroxy-2-oxoglutarate aldolase [EC:4.1.2.14 4.1.3.42] | 1 |
| *S. aureus* | Lumen | Donor | K01689 | ENO1_2_3, eno; enolase 1/2/3 [EC:4.2.1.11] | 1 |
| *S. aureus* | Lumen | Donor | K09458 | fabF, OXSM, CEM1; 3-oxoacyl-[acyl-carrier-protein] synthase II [EC:2.3.1.179] | 1 |
| *S. aureus* | Lumen | Donor | K00208 | fabI; enoyl-[acyl-carrier protein] reductase I [EC:1.3.1.9 1.3.1.10] | 1 |
| *S. aureus* | Lumen | Donor | K03522 | fixB, etfA; electron transfer flavoprotein alpha subunit | 1 |
| *S. aureus* | Lumen | Donor | K00134 | GAPDH, gapA; glyceraldehyde 3-phosphate dehydrogenase (phosphorylating) [EC:1.2.1.12] | 1 |
| *S. aureus* | Lumen | Donor | K02435 | gatC, GATC; aspartyl-tRNA(Asn)/glutamyl-tRNA(Gln) amidotransferase subunit C [EC:6.3.5.6 6.3.5.7] | 1 |
| *S. aureus* | Lumen | Donor | K00252 | GCDH, gcdH; glutaryl-CoA dehydrogenase [EC:1.3.8.6] | 1 |
| *S. aureus* | Lumen | Donor | K01915 | glnA, GLUL; glutamine synthetase [EC:6.3.1.2] | 1 |
| *S. aureus* | Lumen | Donor | K00053 | ilvC; ketol-acid reductoisomerase [EC:1.1.1.86] | 1 |
| *S. aureus* | Lumen | Donor | K01687 | ilvD; dihydroxy-acid dehydratase [EC:4.2.1.9] | 1 |
| *S. aureus* | Lumen | Donor | K11900 | impC; type VI secretion system protein ImpC | 1 |
| *S. aureus* | Lumen | Donor | K04488 | iscU, nifU; nitrogen fixation protein NifU and related proteins | 1 |
| *S. aureus* | Lumen | Donor | K07033 | K07033; uncharacterized protein | 1 |
| *S. aureus* | Lumen | Donor | K07667 | kdpE; two-component system, OmpR family, KDP operon response regulator KdpE | 1 |
| *S. aureus* | Lumen | Donor | K00101 | lldD; L-lactate dehydrogenase (cytochrome) [EC:1.1.2.3] | 1 |
| *S. aureus* | Lumen | Donor | K05606 | MCEE, epi; methylmalonyl-CoA/ethylmalonyl-CoA epimerase [EC:5.1.99.1] | 1 |
| *S. aureus* | Lumen | Donor | K21636 | nrdD; ribonucleoside-triphosphate reductase (formate) [EC:1.1.98.6] | 3 |
| *S. aureus* | Lumen | Donor | Others | - | 48 |
| *S. aureus* | Lumen | Donor | K00927 | PGK, pgk; phosphoglycerate kinase [EC:2.7.2.3] | 1 |
| *S. aureus* | Lumen | Donor | K05781 | phnK; putative phosphonate transport system ATP-binding protein | 1 |
| *S. aureus* | Lumen | Donor | K03386 | PRDX2_4, ahpC; peroxiredoxin 2/4 [EC:1.11.1.24] | 1 |
| *S. aureus* | Lumen | Donor | K09788 | prpF; 2-methylaconitate isomerase [EC:5.3.3.-] | 1 |
| *S. aureus* | Lumen | Donor | K01923 | purC; phosphoribosylaminoimidazole-succinocarboxamide synthase [EC:6.3.2.6] | 1 |
| *S. aureus* | Lumen | Donor | K00610 | pyrI; aspartate carbamoyltransferase regulatory subunit | 1 |
| *S. aureus* | Lumen | Donor | K01779 | racD; aspartate racemase [EC:5.1.1.13] | 1 |
| *S. aureus* | Lumen | Donor | K03553 | recA; recombination protein RecA | 1 |
| *S. aureus* | Lumen | Donor | K01629 | rhaD; rhamnulose-1-phosphate aldolase [EC:4.1.2.19] | 1 |
| *S. aureus* | Lumen | Donor | K09022 | ridA, tdcF, RIDA; 2-iminobutanoate/2-iminopropanoate deaminase [EC:3.5.99.10] | 1 |
| *S. aureus* | Lumen | Donor | K03617 | rnfA; H+/Na+-translocating ferredoxin:NAD+ oxidoreductase subunit A | 1 |
| *S. aureus* | Lumen | Donor | K02867 | RP-L11, MRPL11, rplK; large subunit ribosomal protein L11 | 1 |
| *S. aureus* | Lumen | Donor | K02935 | RP-L7, MRPL12, rplL; large subunit ribosomal protein L7/L12 | 1 |
| *S. aureus* | Lumen | Donor | K02948 | RP-S11, MRPS11, rpsK; small subunit ribosomal protein S11 | 1 |
| *S. aureus* | Lumen | Donor | K02967 | RP-S2, MRPS2, rpsB; small subunit ribosomal protein S2 | 1 |
| *S. aureus* | Lumen | Donor | K02996 | RP-S9, MRPS9, rpsI; small subunit ribosomal protein S9 | 1 |
| *S. aureus* | Lumen | Donor | K00058 | serA, PHGDH; D-3-phosphoglycerate dehydrogenase / 2-oxoglutarate reductase [EC:1.1.1.95 1.1.1.399] | 1 |
| *S. aureus* | Lumen | Donor | K03327 | SLC47A, MATE, DTX; MATE family, multidrug and toxin extrusion protein | 1 |
| *S. aureus* | Lumen | Donor | K09013 | sufC; Fe-S cluster assembly ATP-binding protein | 1 |
| *S. aureus* | Lumen | Donor | K00616 | TALDO1, talB, talA; transaldolase [EC:2.2.1.2] | 1 |
| *S. aureus* | Lumen | Donor | K03299 | TC.GNTP; gluconate:H+ symporter, GntP family | 2 |
| *S. aureus* | Lumen | Donor | K00384 | trxB, TRR; thioredoxin reductase (NADPH) [EC:1.8.1.9] | 1 |
| *S. aureus* | Lumen | Donor | K02377 | TSTA3, fcl; GDP-L-fucose synthase [EC:1.1.1.271] | 1 |
| *S. aureus* | Lumen | Donor | K04085 | tusA, sirA; tRNA 2-thiouridine synthesizing protein A [EC:2.8.1.-] | 1 |
| *S. aureus* | Lumen | Donor | K03476 | ulaG; L-ascorbate 6-phosphate lactonase [EC:3.1.1.-] | 2 |
| *S. aureus* | Lumen | Donor | K11751 | ushA; 5'-nucleotidase / UDP-sugar diphosphatase [EC:3.1.3.5 3.6.1.45] | 1 |
| *S. aureus* | Lumen | Donor | K19068 | wbjC; UDP-2-acetamido-2,6-beta-L-arabino-hexul-4-ose reductase [EC:1.1.1.367] | 1 |
| *S. aureus* | Lumen | Donor | K01791 | wecB; UDP-N-acetylglucosamine 2-epimerase (non-hydrolysing) [EC:5.1.3.14] | 1 |
| *S. aureus* | Lumen | Donor | K01805 | xylA; xylose isomerase [EC:5.3.1.5] | 1 |
| *S. aureus* | Lumen | Donor | K14977 | ylbA, UGHY; (S)-ureidoglycine aminohydrolase [EC:3.5.3.26] | 1 |
| *S. aureus* | Lumen | Recipient | K02003 | ABC.CD.A; putative ABC transport system ATP-binding protein | 1 |
| *S. aureus* | Lumen | Recipient | K02026 | ABC.MS.P1; multiple sugar transport system permease protein | 1 |
| *S. aureus* | Lumen | Recipient | K00926 | arcC; carbamate kinase [EC:2.7.2.2] | 1 |
| *S. aureus* | Lumen | Recipient | K01744 | aspA; aspartate ammonia-lyase [EC:4.3.1.1] | 1 |
| *S. aureus* | Lumen | Recipient | K01646 | citD; citrate lyase subunit gamma (acyl carrier protein) | 1 |
| *S. aureus* | Lumen | Recipient | K03518 | coxS; aerobic carbon-monoxide dehydrogenase small subunit [EC:1.2.5.3] | 1 |
| *S. aureus* | Lumen | Recipient | K04043 | dnaK, HSPA9; molecular chaperone DnaK | 1 |
| *S. aureus* | Lumen | Recipient | K01222 | E3.2.1.86A, celF; 6-phospho-beta-glucosidase [EC:3.2.1.86] | 1 |
| *S. aureus* | Lumen | Recipient | K01625 | eda; 2-dehydro-3-deoxyphosphogluconate aldolase / (4S)-4-hydroxy-2-oxoglutarate aldolase [EC:4.1.2.14 4.1.3.42] | 1 |
| *S. aureus* | Lumen | Recipient | K01889 | FARSA, pheS; phenylalanyl-tRNA synthetase alpha chain [EC:6.1.1.20] | 1 |
| *S. aureus* | Lumen | Recipient | K02355 | fusA, GFM, EFG; elongation factor G | 1 |
| *S. aureus* | Lumen | Recipient | K01878 | glyQ; glycyl-tRNA synthetase alpha chain [EC:6.1.1.14] | 1 |
| *S. aureus* | Lumen | Recipient | K15633 | gpmI; 2,3-bisphosphoglycerate-independent phosphoglycerate mutase [EC:5.4.2.12] | 1 |
| *S. aureus* | Lumen | Recipient | K04077 | groEL, HSPD1; chaperonin GroEL [EC:5.6.1.7] | 1 |
| *S. aureus* | Lumen | Recipient | K01951 | guaA, GMPS; GMP synthase (glutamine-hydrolysing) [EC:6.3.5.2] | 1 |
| *S. aureus* | Lumen | Recipient | K01687 | ilvD; dihydroxy-acid dehydratase [EC:4.2.1.9] | 1 |
| *S. aureus* | Lumen | Recipient | K02518 | infA; translation initiation factor IF-1 | 1 |
| *S. aureus* | Lumen | Recipient | K00874 | kdgK; 2-dehydro-3-deoxygluconokinase [EC:2.7.1.45] | 1 |
| *S. aureus* | Lumen | Recipient | K00290 | LYS1; saccharopine dehydrogenase (NAD+, L-lysine forming) [EC:1.5.1.7] | 1 |
| *S. aureus* | Lumen | Recipient | K07171 | mazF, ndoA, chpA; mRNA interferase MazF [EC:3.1.-.-] | 1 |
| *S. aureus* | Lumen | Recipient | K01740 | metY; O-acetylhomoserine (thiol)-lyase [EC:2.5.1.49] | 2 |
| *S. aureus* | Lumen | Recipient | K02567 | napA; nitrate reductase (cytochrome) [EC:1.9.6.1] | 1 |
| *S. aureus* | Lumen | Recipient | K21636 | nrdD; ribonucleoside-triphosphate reductase (formate) [EC:1.1.98.6] | 1 |
| *S. aureus* | Lumen | Recipient | K10254 | ohyA, sph; oleate hydratase [EC:4.2.1.53] | 1 |
| *S. aureus* | Lumen | Recipient | Others | - | 15 |
| *S. aureus* | Lumen | Recipient | K00074 | paaH, hbd, fadB, mmgB; 3-hydroxybutyryl-CoA dehydrogenase [EC:1.1.1.157] | 1 |
| *S. aureus* | Lumen | Recipient | K01610 | pckA; phosphoenolpyruvate carboxykinase (ATP) [EC:4.1.1.49] | 2 |
| *S. aureus* | Lumen | Recipient | K05781 | phnK; putative phosphonate transport system ATP-binding protein | 2 |
| *S. aureus* | Lumen | Recipient | K01006 | ppdK; pyruvate, orthophosphate dikinase [EC:2.7.9.1] | 1 |
| *S. aureus* | Lumen | Recipient | K03386 | PRDX2_4, ahpC; peroxiredoxin 2/4 [EC:1.11.1.24] | 1 |
| *S. aureus* | Lumen | Recipient | K02837 | prfC; peptide chain release factor 3 | 1 |
| *S. aureus* | Lumen | Recipient | K01933 | purM; phosphoribosylformylglycinamidine cyclo-ligase [EC:6.3.3.1] | 2 |
| *S. aureus* | Lumen | Recipient | K02871 | RP-L13, MRPL13, rplM; large subunit ribosomal protein L13 | 1 |
| *S. aureus* | Lumen | Recipient | K02874 | RP-L14, MRPL14, rplN; large subunit ribosomal protein L14 | 1 |
| *S. aureus* | Lumen | Recipient | K02876 | RP-L15, MRPL15, rplO; large subunit ribosomal protein L15 | 1 |
| *S. aureus* | Lumen | Recipient | K02887 | RP-L20, MRPL20, rplT; large subunit ribosomal protein L20 | 2 |
| *S. aureus* | Lumen | Recipient | K02935 | RP-L7, MRPL12, rplL; large subunit ribosomal protein L7/L12 | 2 |
| *S. aureus* | Lumen | Recipient | K02961 | RP-S17, MRPS17, rpsQ; small subunit ribosomal protein S17 | 1 |
| *S. aureus* | Lumen | Recipient | K00058 | serA, PHGDH; D-3-phosphoglycerate dehydrogenase / 2-oxoglutarate reductase [EC:1.1.1.95 1.1.1.399] | 1 |
| *S. aureus* | Lumen | Recipient | K06412 | spoVG; stage V sporulation protein G | 1 |
| *S. aureus* | Lumen | Recipient | K09693 | tagH; teichoic acid transport system ATP-binding protein [EC:7.5.2.4] | 1 |
| *S. aureus* | Lumen | Recipient | K03451 | TC.BCT; betaine/carnitine transporter, BCCT family | 1 |
| *S. aureus* | Lumen | Recipient | K03299 | TC.GNTP; gluconate:H+ symporter, GntP family | 1 |
| *S. aureus* | Lumen | Recipient | K06925 | tsaE; tRNA threonylcarbamoyladenosine biosynthesis protein TsaE | 1 |
| *S. aureus* | Lumen | Recipient | K02357 | tsf, TSFM; elongation factor Ts | 1 |
| *S. aureus* | Lumen | Recipient | K08234 | yaeR; glyoxylase I family protein | 1 |
| CIP | Lumen | Donor | K04043 | dnaK, HSPA9; molecular chaperone DnaK | 1 |
| CIP | Lumen | Donor | K02355 | fusA, GFM, EFG; elongation factor G | 1 |
| CIP | Lumen | Donor | K00600 | glyA, SHMT; glycine hydroxymethyltransferase [EC:2.1.2.1] | 1 |
| CIP | Lumen | Donor | K01878 | glyQ; glycyl-tRNA synthetase alpha chain [EC:6.1.1.14] | 1 |
| CIP | Lumen | Donor | K01711 | gmd, GMDS; GDPmannose 4,6-dehydratase [EC:4.2.1.47] | 1 |
| CIP | Lumen | Donor | K15633 | gpmI; 2,3-bisphosphoglycerate-independent phosphoglycerate mutase [EC:5.4.2.12] | 1 |
| CIP | Lumen | Donor | K01951 | guaA, GMPS; GMP synthase (glutamine-hydrolysing) [EC:6.3.5.2] | 1 |
| CIP | Lumen | Donor | K02518 | infA; translation initiation factor IF-1 | 1 |
| CIP | Lumen | Donor | K04567 | KARS, lysS; lysyl-tRNA synthetase, class II [EC:6.1.1.6] | 1 |
| CIP | Lumen | Donor | K00874 | kdgK; 2-dehydro-3-deoxygluconokinase [EC:2.7.1.45] | 1 |
| CIP | Lumen | Donor | K00290 | LYS1; saccharopine dehydrogenase (NAD+, L-lysine forming) [EC:1.5.1.7] | 1 |
| CIP | Lumen | Donor | K00789 | metK, MAT; S-adenosylmethionine synthetase [EC:2.5.1.6] | 1 |
| CIP | Lumen | Donor | K01847 | MUT; methylmalonyl-CoA mutase [EC:5.4.99.2] | 1 |
| CIP | Lumen | Donor | K00278 | nadB; L-aspartate oxidase [EC:1.4.3.16] | 1 |
| CIP | Lumen | Donor | K00349 | nqrD; Na+-transporting NADH:ubiquinone oxidoreductase subunit D [EC:7.2.1.1] | 1 |
| CIP | Lumen | Donor | Others | - | 7 |
| CIP | Lumen | Donor | K01610 | pckA; phosphoenolpyruvate carboxykinase (ATP) [EC:4.1.1.49] | 1 |
| CIP | Lumen | Donor | K03386 | PRDX2_4, ahpC; peroxiredoxin 2/4 [EC:1.11.1.24] | 2 |
| CIP | Lumen | Donor | K02837 | prfC; peptide chain release factor 3 | 1 |
| CIP | Lumen | Donor | K01933 | purM; phosphoribosylformylglycinamidine cyclo-ligase [EC:6.3.3.1] | 1 |
| CIP | Lumen | Donor | K09457 | queF; 7-cyano-7-deazaguanine reductase [EC:1.7.1.13] | 1 |
| CIP | Lumen | Donor | K02871 | RP-L13, MRPL13, rplM; large subunit ribosomal protein L13 | 1 |
| CIP | Lumen | Donor | K02876 | RP-L15, MRPL15, rplO; large subunit ribosomal protein L15 | 1 |
| CIP | Lumen | Donor | K02887 | RP-L20, MRPL20, rplT; large subunit ribosomal protein L20 | 2 |
| CIP | Lumen | Donor | K02888 | RP-L21, MRPL21, rplU; large subunit ribosomal protein L21 | 1 |
| CIP | Lumen | Donor | K02935 | RP-L7, MRPL12, rplL; large subunit ribosomal protein L7/L12 | 1 |
| CIP | Lumen | Donor | K02963 | RP-S18, MRPS18, rpsR; small subunit ribosomal protein S18 | 1 |
| CIP | Lumen | Donor | K00058 | serA, PHGDH; D-3-phosphoglycerate dehydrogenase / 2-oxoglutarate reductase [EC:1.1.1.95 1.1.1.399] | 1 |
| CIP | Lumen | Donor | K01585 | speA; arginine decarboxylase [EC:4.1.1.19] | 1 |
| CIP | Lumen | Donor | K03150 | thiH; 2-iminoacetate synthase [EC:4.1.99.19] | 1 |
| CIP | Lumen | Donor | K06925 | tsaE; tRNA threonylcarbamoyladenosine biosynthesis protein TsaE | 1 |
| CIP | Lumen | Recipient | K03741 | arsC; arsenate reductase (thioredoxin) [EC:1.20.4.4] | 1 |
| CIP | Lumen | Recipient | K02112 | ATPF1B, atpD; F-type H+/Na+-transporting ATPase subunit beta [EC:7.1.2.2 7.2.2.1] | 1 |
| CIP | Lumen | Recipient | K04043 | dnaK, HSPA9; molecular chaperone DnaK | 1 |
| CIP | Lumen | Recipient | K07405 | E3.2.1.1A; alpha-amylase [EC:3.2.1.1] | 1 |
| CIP | Lumen | Recipient | K01639 | E4.1.3.3, nanA, NPL; N-acetylneuraminate lyase [EC:4.1.3.3] | 1 |
| CIP | Lumen | Recipient | K01678 | E4.2.1.2AB, fumB; fumarate hydratase subunit beta [EC:4.2.1.2] | 1 |
| CIP | Lumen | Recipient | K01848 | E5.4.99.2A, mcmA1; methylmalonyl-CoA mutase, N-terminal domain [EC:5.4.99.2] | 1 |
| CIP | Lumen | Recipient | K01849 | E5.4.99.2B, mcmA2; methylmalonyl-CoA mutase, C-terminal domain [EC:5.4.99.2] | 1 |
| CIP | Lumen | Recipient | K01625 | eda; 2-dehydro-3-deoxyphosphogluconate aldolase / (4S)-4-hydroxy-2-oxoglutarate aldolase [EC:4.1.2.14 4.1.3.42] | 1 |
| CIP | Lumen | Recipient | K00208 | fabI; enoyl-[acyl-carrier protein] reductase I [EC:1.3.1.9 1.3.1.10] | 1 |
| CIP | Lumen | Recipient | K03522 | fixB, etfA; electron transfer flavoprotein alpha subunit | 1 |
| CIP | Lumen | Recipient | K00134 | GAPDH, gapA; glyceraldehyde 3-phosphate dehydrogenase (phosphorylating) [EC:1.2.1.12] | 1 |
| CIP | Lumen | Recipient | K00046 | idnO; gluconate 5-dehydrogenase [EC:1.1.1.69] | 1 |
| CIP | Lumen | Recipient | K05606 | MCEE, epi; methylmalonyl-CoA/ethylmalonyl-CoA epimerase [EC:5.1.99.1] | 1 |
| CIP | Lumen | Recipient | K01740 | metY; O-acetylhomoserine (thiol)-lyase [EC:2.5.1.49] | 1 |
| CIP | Lumen | Recipient | K00278 | nadB; L-aspartate oxidase [EC:1.4.3.16] | 1 |
| CIP | Lumen | Recipient | Others | - | 16 |
| CIP | Lumen | Recipient | K01610 | pckA; phosphoenolpyruvate carboxykinase (ATP) [EC:4.1.1.49] | 1 |
| CIP | Lumen | Recipient | K13668 | pimB; phosphatidyl-myo-inositol dimannoside synthase [EC:2.4.1.346] | 1 |
| CIP | Lumen | Recipient | K03737 | por, nifJ; pyruvate-ferredoxin/flavodoxin oxidoreductase [EC:1.2.7.1 1.2.7.-] | 1 |
| CIP | Lumen | Recipient | K01923 | purC; phosphoribosylaminoimidazole-succinocarboxamide synthase [EC:6.3.2.6] | 2 |
| CIP | Lumen | Recipient | K01933 | purM; phosphoribosylformylglycinamidine cyclo-ligase [EC:6.3.3.1] | 1 |
| CIP | Lumen | Recipient | K01591 | pyrF; orotidine-5'-phosphate decarboxylase [EC:4.1.1.23] | 1 |
| CIP | Lumen | Recipient | K01886 | QARS, glnS; glutaminyl-tRNA synthetase [EC:6.1.1.18] | 1 |
| CIP | Lumen | Recipient | K09765 | queH; epoxyqueuosine reductase [EC:1.17.99.6] | 1 |
| CIP | Lumen | Recipient | K01813 | rhaA; L-rhamnose isomerase [EC:5.3.1.14] | 1 |
| CIP | Lumen | Recipient | K02867 | RP-L11, MRPL11, rplK; large subunit ribosomal protein L11 | 2 |
| CIP | Lumen | Recipient | K02887 | RP-L20, MRPL20, rplT; large subunit ribosomal protein L20 | 1 |
| CIP | Lumen | Recipient | K02888 | RP-L21, MRPL21, rplU; large subunit ribosomal protein L21 | 1 |
| CIP | Lumen | Recipient | K02935 | RP-L7, MRPL12, rplL; large subunit ribosomal protein L7/L12 | 1 |
| CIP | Lumen | Recipient | K02945 | RP-S1, rpsA; small subunit ribosomal protein S1 | 1 |
| CIP | Lumen | Recipient | K02948 | RP-S11, MRPS11, rpsK; small subunit ribosomal protein S11 | 1 |
| CIP | Lumen | Recipient | K02963 | RP-S18, MRPS18, rpsR; small subunit ribosomal protein S18 | 2 |
| CIP | Lumen | Recipient | K02967 | RP-S2, MRPS2, rpsB; small subunit ribosomal protein S2 | 1 |
| CIP | Lumen | Recipient | K02996 | RP-S9, MRPS9, rpsI; small subunit ribosomal protein S9 | 1 |
| CIP | Lumen | Recipient | K00239 | sdhA, frdA; succinate dehydrogenase flavoprotein subunit [EC:1.3.5.1] | 1 |
| CIP | Lumen | Recipient | K00058 | serA, PHGDH; D-3-phosphoglycerate dehydrogenase / 2-oxoglutarate reductase [EC:1.1.1.95 1.1.1.399] | 1 |
| CIP | Lumen | Recipient | K03149 | thiG; thiazole synthase [EC:2.8.1.10] | 1 |
| CIP | Lumen | Recipient | K01805 | xylA; xylose isomerase [EC:5.3.1.5] | 1 |
| Co-exposure | Lumen | Donor | K18369 | adh2; alcohol dehydrogenase [EC:1.1.1.-] | 1 |
| Co-exposure | Lumen | Donor | K00046 | idnO; gluconate 5-dehydrogenase [EC:1.1.1.69] | 1 |
| Co-exposure | Lumen | Donor | Others | - | 4 |
| Co-exposure | Lumen | Donor | K01610 | pckA; phosphoenolpyruvate carboxykinase (ATP) [EC:4.1.1.49] | 1 |
| Co-exposure | Lumen | Donor | K00602 | purH; phosphoribosylaminoimidazolecarboxamide formyltransferase / IMP cyclohydrolase [EC:2.1.2.3 3.5.4.10] | 1 |
| Co-exposure | Lumen | Donor | K01813 | rhaA; L-rhamnose isomerase [EC:5.3.1.14] | 1 |
| Co-exposure | Lumen | Donor | K02867 | RP-L11, MRPL11, rplK; large subunit ribosomal protein L11 | 1 |
| Co-exposure | Lumen | Donor | K02887 | RP-L20, MRPL20, rplT; large subunit ribosomal protein L20 | 1 |
| Co-exposure | Lumen | Donor | K02935 | RP-L7, MRPL12, rplL; large subunit ribosomal protein L7/L12 | 1 |
| Co-exposure | Lumen | Donor | K02961 | RP-S17, MRPS17, rpsQ; small subunit ribosomal protein S17 | 1 |
| Co-exposure | Lumen | Donor | K03476 | ulaG; L-ascorbate 6-phosphate lactonase [EC:3.1.1.-] | 1 |
| Co-exposure | Lumen | Donor | K07322 | ytfE, scdA; regulator of cell morphogenesis and NO signaling | 1 |
| Co-exposure | Lumen | Recipient | K01711 | gmd, GMDS; GDPmannose 4,6-dehydratase [EC:4.2.1.47] | 1 |
| Co-exposure | Lumen | Recipient | Others | - | 2 |
| *S. aureus* | Mucosa | Donor | K02013 | ABC.FEV.A; iron complex transport system ATP-binding protein [EC:7.2.2.-] | 1 |
| *S. aureus* | Mucosa | Donor | K10542 | mglA; methyl-galactoside transport system ATP-binding protein [EC:7.5.2.11] | 1 |
| *S. aureus* | Mucosa | Donor | Others | - | 1 |
| *S. aureus* | Mucosa | Recipient | K02003 | ABC.CD.A; putative ABC transport system ATP-binding protein | 1 |
| *S. aureus* | Mucosa | Recipient | K01740 | metY; O-acetylhomoserine (thiol)-lyase [EC:2.5.1.49] | 1 |
| *S. aureus* | Mucosa | Recipient | Others | - | 1 |
| CIP | Mucosa | Donor | K02003 | ABC.CD.A; putative ABC transport system ATP-binding protein | 1 |
| CIP | Mucosa | Donor | K02013 | ABC.FEV.A; iron complex transport system ATP-binding protein [EC:7.2.2.-] | 2 |
| CIP | Mucosa | Donor | K00600 | glyA, SHMT; glycine hydroxymethyltransferase [EC:2.1.2.1] | 1 |
| CIP | Mucosa | Donor | K04567 | KARS, lysS; lysyl-tRNA synthetase, class II [EC:6.1.1.6] | 1 |
| CIP | Mucosa | Donor | K00789 | metK, MAT; S-adenosylmethionine synthetase [EC:2.5.1.6] | 1 |
| CIP | Mucosa | Donor | K00349 | nqrD; Na+-transporting NADH:ubiquinone oxidoreductase subunit D [EC:7.2.1.1] | 1 |
| CIP | Mucosa | Donor | Others | - | 4 |
| CIP | Mucosa | Donor | K05781 | phnK; putative phosphonate transport system ATP-binding protein | 1 |
| CIP | Mucosa | Donor | K02871 | RP-L13, MRPL13, rplM; large subunit ribosomal protein L13 | 1 |
| CIP | Mucosa | Recipient | K02056 | ABC.SS.A; simple sugar transport system ATP-binding protein [EC:7.5.2.-] | 1 |
| CIP | Mucosa | Recipient | K10907 | K10907; aminotransferase [EC:2.6.1.-] | 1 |
| CIP | Mucosa | Recipient | Others | - | 4 |
| CIP | Mucosa | Recipient | K05781 | phnK; putative phosphonate transport system ATP-binding protein | 2 |
| CIP | Mucosa | Recipient | K03476 | ulaG; L-ascorbate 6-phosphate lactonase [EC:3.1.1.-] | 1 |
| Co-exposure | Mucosa | Donor | K07033 | K07033; uncharacterized protein | 1 |
| Co-exposure | Mucosa | Donor | K01996 | livF; branched-chain amino acid transport system ATP-binding protein | 1 |
| Co-exposure | Mucosa | Donor | K01995 | livG; branched-chain amino acid transport system ATP-binding protein | 1 |
| Co-exposure | Mucosa | Donor | K01740 | metY; O-acetylhomoserine (thiol)-lyase [EC:2.5.1.49] | 1 |
| Co-exposure | Mucosa | Donor | K03476 | ulaG; L-ascorbate 6-phosphate lactonase [EC:3.1.1.-] | 1 |
| Co-exposure | Mucosa | Recipient | K02013 | ABC.FEV.A; iron complex transport system ATP-binding protein [EC:7.2.2.-] | 3 |
| Co-exposure | Mucosa | Recipient | K01996 | livF; branched-chain amino acid transport system ATP-binding protein | 2 |

**References**

1. Zhou W, Yuan X, Long A, Huang H, Yue W. Different hydrodynamic processes regulated on water quality (nutrients, dissolved oxygen, and phytoplankton biomass) in three contrasting waters of Hong Kong. Environ Monit Assess 2014; 186:1705-18.

2. CLSI. Performance standards for antimicrobial susceptibility testing, M100, 31st ed.: Clinical Laboratory Standards Institute, 2021.

3. Missiakas DM, Schneewind O. Growth and laboratory maintenance of Staphylococcus aureus. Curr Protoc Microbiol 2013; 28:9C. 1.-9C. 1.9.

4. Dirtu AC, Covaci A. Estimation of daily intake of organohalogenated contaminants from food consumption and indoor dust ingestion in Romania. Environmental science & technology 2010; 44:6297-304.

5. Guo J, Wu F, Shen R, Zeng EY. Dietary intake and potential health risk of DDTs and PBDEs via seafood consumption in South China. Ecotoxicol Environ Saf 2010; 73:1812-9.

6. Liu X, Steele JC, Meng X-Z. Usage, residue, and human health risk of antibiotics in Chinese aquaculture: A review. Environ Pollut 2017; 223:161-9.

7. Ezzeldeen NA, Mansour HA, Ahmed AA. Phenotypic and molecular identification of Staphylococcus aureus isolated from some Egyptian salted fish. World Appl Sci J 2011; 15:1703-12.

8. Herren CM, McMahon KD. Cohesion: a method for quantifying the connectivity of microbial communities. ISME J 2017; 11:2426-38.

9. Wu D, Jin L, Xie J, Liu H, Zhao J, Ye D, et al. Inhalable antibiotic resistomes emitted from hospitals: metagenomic insights into bacterial hosts, clinical relevance, and environmental risks. Microbiome 2022; 10:19.

10. Cantalapiedra CP, Hernández-Plaza A, Letunic I, Bork P, Huerta-Cepas J. eggNOG-mapper v2: functional annotation, orthology assignments, and domain prediction at the metagenomic scale. Mol Biol Evol 2021; 38:5825-9.

11. Bao Y, Ruan Y, Wu J, Wang W-X, Leung KM, Lee PK. Metagenomics-Based Microbial Ecological Community Threshold and Indicators of Anthropogenic Disturbances in Estuarine Sediments. Environ Sci Technol 2023; 58:780-94.

12. Malik AA, Martiny JB, Brodie EL, Martiny AC, Treseder KK, Allison SD. Defining trait-based microbial strategies with consequences for soil carbon cycling under climate change. ISME J 2020; 14:1-9.

13. Watts SC, Ritchie SC, Inouye M, Holt KE. FastSpar: rapid and scalable correlation estimation for compositional data. Bioinform 2019; 35:1064-6.

14. Weiss S, Van Treuren W, Lozupone C, Faust K, Friedman J, Deng Y, et al. Correlation detection strategies in microbial data sets vary widely in sensitivity and precision. ISME J 2016; 10:1669-81.

15. Wu H, Gao T, Hu A, Wang J. Network Complexity and Stability of Microbes Enhanced by Microplastic Diversity. Environ Sci Technol 2024; 58:4334-45.

16. Yuan MM, Guo X, Wu L, Zhang Y, Xiao N, Ning D, et al. Climate warming enhances microbial network complexity and stability. Nat Clim Chang 2021; 11:343-8.

17. Chen S, Zhou Y, Chen Y, Gu J. fastp: an ultra-fast all-in-one FASTQ preprocessor. Bioinformatics 2018; 34:i884-i90.

18. Wood DE, Lu J, Langmead B. Improved metagenomic analysis with Kraken 2. Genome Biol 2019; 20:1-13.

19. Lu J, Breitwieser FP, Thielen P, Salzberg SL. Bracken: estimating species abundance in metagenomics data. PeerJ Computer science 2017; 3:e104.

20. Arango-Argoty G, Garner E, Pruden A, Heath LS, Vikesland P, Zhang L. DeepARG: a deep learning approach for predicting antibiotic resistance genes from metagenomic data. Microbiome 2018; 6:1-15.

21. Tatusova T, DiCuccio M, Badretdin A, Chetvernin V, Nawrocki EP, Zaslavsky L, et al. NCBI prokaryotic genome annotation pipeline. Nucleic Acids Res 2016; 44:6614-24.

22. Uritskiy GV, DiRuggiero J, Taylor J. MetaWRAP—a flexible pipeline for genome-resolved metagenomic data analysis. Microbiome 2018; 6:1-13.

23. Kim D, Song L, Breitwieser FP, Salzberg SL. Centrifuge: rapid and sensitive classification of metagenomic sequences. Genome Res 2016; 26:1721-9.

24. Feng Y, Wang Y, Zhu B, Gao GF, Guo Y, Hu Y. Metagenome-assembled genomes and gene catalog from the chicken gut microbiome aid in deciphering antibiotic resistomes. Commun Biol 2021; 4:1305.

25. Parks DH, Imelfort M, Skennerton CT, Hugenholtz P, Tyson GW. CheckM: assessing the quality of microbial genomes recovered from isolates, single cells, and metagenomes. Genome Res 2015; 25:1043-55.

26. Olm MR, Brown CT, Brooks B, Banfield JF. dRep: a tool for fast and accurate genomic comparisons that enables improved genome recovery from metagenomes through de-replication. ISME J 2017; 11:2864-8.

27. Hyatt D, Chen G-L, LoCascio PF, Land ML, Larimer FW, Hauser LJ. Prodigal: prokaryotic gene recognition and translation initiation site identification. BMC Bioinformatics 2010; 11:1-11.

28. Fu L, Niu B, Zhu Z, Wu S, Li W. CD-HIT: accelerated for clustering the next-generation sequencing data. Bioinform 2012; 28:3150-2.

29. Finn RD, Clements J, Eddy SR. HMMER web server: interactive sequence similarity searching. Nucleic Acids Res 2011; 39:W29-W37.

30. Enault F, Briet A, Bouteille L, Roux S, Sullivan MB, Petit MA. Phages rarely encode antibiotic resistance genes: a cautionary tale for virome analyses. ISME J 2017; 11:237-47.

31. Leung MHY, Tong X, Boifot KO, Bezdan D, Butler DJ, Danko DC, et al. Characterization of the public transit air microbiome and resistome reveals geographical specificity. Microbiome 2021; 9:112.

32. Song W, Wemheuer B, Zhang S, Steensen K, Thomas T. MetaCHIP: community-level horizontal gene transfer identification through the combination of best-match and phylogenetic approaches. Microbiome 2019; 7:1-14.

33. Letunic I, Bork P. Interactive Tree Of Life (iTOL) v5: an online tool for phylogenetic tree display and annotation. Nucleic Acids Res 2021; 49:W293-W6.

34. Taiaroa G, Matalavea B, Tafuna'i M, Lacey JA, Price DJ, Isaia L, et al. Scabies and impetigo in Samoa: a school-based clinical and molecular epidemiological study. Lancet Reg Health West Pac 2021; 6.
